# Phenotypic Heterogeneity of Genomically-Diverse Isolates of *Streptococcus mutans*

**DOI:** 10.1371/journal.pone.0061358

**Published:** 2013-04-16

**Authors:** Sara R. Palmer, James H. Miller, Jacqueline Abranches, Lin Zeng, Tristan Lefebure, Vincent P. Richards, José A. Lemos, Michael J. Stanhope, Robert A. Burne

**Affiliations:** 1 Department of Oral Biology, University of Florida, Gainesville, Florida, United States of America; 2 Université de Lyon, CNRS, Ecologie des Hydrosystèmes Naturels et Anthropisés; Université Lyon, Villeurbanne, France; 3 Population Medicine and Diagnostic Sciences, College of Veterinary Medicine, Cornell University, Ithaca, New York, United States of America; 4 Center for Oral Biology, University of Rochester Medical Center, Rochester, New York, United States of America; 5 Department of Microbiology and Immunology, University of Rochester Medical Center, Rochester, New York, United States of America; University of Kansas Medical Center, United States of America

## Abstract

High coverage, whole genome shotgun (WGS) sequencing of 57 geographically- and genetically-diverse isolates of *Streptococcus mutans* from individuals of known dental caries status was recently completed. Of the 57 sequenced strains, fifteen isolates, were selected based primarily on differences in gene content and phenotypic characteristics known to affect virulence and compared with the reference strain UA159. A high degree of variability in these properties was observed between strains, with a broad spectrum of sensitivities to low pH, oxidative stress (air and paraquat) and exposure to competence stimulating peptide (CSP). Significant differences in autolytic behavior and in biofilm development in glucose or sucrose were also observed. Natural genetic competence varied among isolates, and this was correlated to the presence or absence of competence genes, *comCDE* and *comX*, and to bacteriocins. In general strains that lacked the ability to become competent possessed fewer genes for bacteriocins and immunity proteins or contained polymorphic variants of these genes. WGS sequence analysis of the pan-genome revealed, for the first time, components of a Type VII secretion system in several *S. mutans* strains, as well as two putative ORFs that encode possible collagen binding proteins located upstream of the *cnm* gene, which is associated with host cell invasiveness. The virulence of these particular strains was assessed in a wax-worm model. This is the first study to combine a comprehensive analysis of key virulence-related phenotypes with extensive genomic analysis of a pathogen that evolved closely with humans. Our analysis highlights the phenotypic diversity of *S. mutans* isolates and indicates that the species has evolved a variety of adaptive strategies to persist in the human oral cavity and, when conditions are favorable, to initiate disease.

## Introduction

The development of dental caries is a complex process that is primarily dependent on the presence of microbial biofilms, the composition and biochemical activity of the biofilm organisms, and the diet of the host; but is also affected by a variety of other factors that include the genetic constitution and behavior of the host, tooth architecture and exposure to fluoride [1]–[5]. *Streptococcus mutans* has long been acknowledged as the species of bacteria most closely associated with the initiation of dental caries [6], [7]. More recently, epidemiological [8] and mechanistic evidence for associations of certain sub-groups of *S. mutans* with cardiovascular disease have emerged [9], [10]. The three key virulence attributes of *S. mutans* that enable this organism to cause dental caries are the ability to form biofilms on the tooth, mediated by sucrose-dependent and sucrose-independent mechanisms [11]; production of organic acids via metabolism of dietary carbohydrates; and the ability to grow and to continue to produce acids in a low pH environment, known as aciduricity [6], [12]. In addition, the ability of *S. mutans* to rapidly adapt to environmental stresses appears to be central to its ability to form biofilms, persist in the host, and to compete with other oral bacteria, particularly when conditions are conducive to the development of dental caries [13]. Furthermore, some *S. mutans* strains are naturally competent for genetic transformation and are able to take up DNA from their environment [14]. Additionally, the competence pathway of *S. mutans* is linked to the production of bacteriocins, which kill susceptible closely related species, thus eliminating competitors while increasing the genetic material available for homologous recombination [15].

*S. mutans* is a diverse species of bacteria that can usually be classified into four different serological groups (*c*, *e*, *f*, and *k*) based on the composition of cell-surface rhamose-glucose polysaccharides [16]. Most strains isolated from the oral cavity (70–80%) are serotype *c*, with 20% composed of serotype *e* and 2–5% serotype *f* or *k*. However, specimens isolated from heart valves and atheromatous plaques have a higher occurrence of non-serotype *c* strains, with serotype *k* in higher proportions (12%) than in the oral cavity [17]. There have been several attempts to correlate carriage of certain genotypes of *S. mutans* with caries incidence, however there has been no consensus among multiple studies [18]–[23]. Additionally, it has been reported that there was no correlation between the caries status of an individual and the distribution of 41 putative virulence genes or genetic elements in 33 *S. mutans* isolates [24]. These authors [24] concluded that the virulence genes they tested might be part of the core genome of *S. mutans*, hence the lack of diversity in their distribution among strains.

Studies using comparative genomic hybridization (CGH) based on the UA159 genome [25] have shown a high degree of content variation among strains, with some isolates lacking up to 20% of the genes present in the reference strain UA159 [26], [27]. In particular there are variations in the presence and content of a 50-kb genomic island, TnSmu2, that contains genes for non-ribosomal peptide synthases (NRPS), polyketide synthases (PKS), and accessory proteins responsible for biosynthesis of mutanobactin, which appears to augment oxidative stress tolerance [28], [29]. Another study identified 122 sequence types (ST) out of 135 strains isolated from around the world [30] using multi-locus sequence typing (MLST) based on the partial gene sequence of 6 housekeeping genes from *S. mutans* [31]; further demonstrating the genetic diversity of this species and reinforcing the findings that there is not a consistent correlation of the presence of certain genotypes with geographic location or caries status.

While techniques like CGH and MLST, as well as numerous other genetic fingerprinting studies, have allowed researchers to interrogate genotype distribution and to gain an understanding of species diversity, they do not allow for genome-scale correlations of phenotype and genotype, nor can they facilitate functional genomic studies that are key to dissecting how gene content and context relate to the pathogenic potential of the organisms [13], [32]. This is especially true for an organism like *S. mutans,* which can become naturally competent and therefore has the potential for rapid genome diversification through lateral gene transfer [33].

Given the clear evidence for substantial genetic diversity in the species *S. mutans* and to better understand the gene content of this species as a whole (i.e. unique core and dispensable genes), completed draft genomes of 57 geographically- and genetically-diverse isolates of *S. mutans* were generated and analyzed [34]. Based on the sequence information, 15 isolates with a high degree of diversity in gene content were chosen for further phenotypic characterization and genetic analyses. This study represents the first step toward determining whether it is possible to correlate core and pan-genome composition with specific phenotypic characteristics that are associated with the virulence potential of *S. mutans*. The knowledge gained from these studies can be used to guide more detailed analysis, e.g. transcriptomic studies and future epidemiologic work, to facilitate methods for the control of *S. mutans* and other cariogenic bacteria. Further, the baseline information provided here establishes a resource that can be utilized to accelerate progress on *S. mutans* pathogenesis and control of dental caries, as well as certain systemic diseases associated with *S. mutans* and closely-related species.

## Materials and Methods

### Bacterial Strains, Media, and Growth Conditions

Isolates of *S. mutans* used in this study are listed in Table 1. All strains were stored in 25% glycerol at −80°C and freshly streaked on brain heart infusion (BHI) agar before each experiment. Routine cultures of *S. mutans* strains were inoculated from a single colony and grown in brain heart infusion (BHI) broth (Difco) at 37°C in a 5% CO_2_ atmosphere. For biofilm experiments, strains were grown in a semi-defined biofilm medium (BM) [35] supplemented with 20 mM glucose or sucrose. For monitoring of growth, overnight cultures from two separate colonies were sub-cultured 1∶25 into fresh medium, grown to mid-exponential phase (OD_600_ = 0.5), and diluted 1∶100 into fresh growth media: BHI pH 7.5; BHI that had been titrated to pH 5.5 with HCl; BHI containing 0.2 µM synthetic competence stimulating peptide (CSP) [36] or BHI containing 25 mM paraquat (methyl viologen; catalog no. M2254; Sigma). Growth was then monitored by dispensing 200 µl of the diluted cultures in duplicate into wells of a Bioscreen C plate with a sterile mineral oil overlay to reduce exposure to oxygen, unless otherwise indicated. Plates were incubated at 37°C for 24 to 48 h in a Bioscreen C lab system (Helsinki, Finland) with readings every 20 min after shaking for 10 sec. Doubling times were calculated as described elsewhere [37] and Student’s *t*-tests were performed to determine significant differences.

**Table 1:** Clinical isolates used in this study.

Accession #	Lab ID	Strain Name	Host/Sample	Origin/MLST	Serotype
AE014133	Smu159	UA159	Dental plaque	NA	c
AHRJ00000000	Smu20	15JP3	ISOC, CF	Brazil/ST12	c
AHRK00000000	Smu21	1SM1	29 months, ISOC, CF	Brazil/ST13	e
AHRT00000000	Smu44	11VS1	30 months, ISOC, CF	Brazil/ST28	c
AHRV00000000	Smu52	NFSM2	Dental plaque	UK/ST2	c
AHRY00000000	Smu56	N29	Dental plaque	UK/ST11	c
AHRZ00000000	Smu57	NMT4863	Dental plaque	Japan/ST12	c
AHSE00000000	Smu63	T4	CF, SGP	UK/ST23	c
AHSH00000000	Smu69	NLM4	Dental plaque	UK/ST37	e
AHSN00000000	Smu77	(N)V1996	Mutant strain of V403*	US/ST47	c
AHSQ00000000	Smu81	SF14	SGP	US/ST58	c
AHSU00000000	Smu86	U2A	Dental plaque	Turkey/ST69	e
AHSZ00000000	Smu93	21	CA	Iceland/ST73	e
AHTD00000000	Smu98	SM1	SGP	HK/ST116	c
AHRE00000000	Smu104	SA41	SGP	ZA/ST114	c
AHRI00000000	Smu109	OMZ175	ISOC, CF	Switz./NA	f

* V403- human blood isolate from R. Facklam, Centers for Disease Control, Atlanta, Ga [112].

HK- Hong Kong, ZA- South Africa, Switz.- Switzerland. SGP- Supra-gingival plaque, ISOC- initial stages of oral colonization, CF-caries free, CA-caries active.

### Biofilm Assays

Biofilm development was measured in polystyrene 96-well (flat-bottom) cell culture clusters (Costar.595; Corning Inc., Corning, NY) as previously described [38], with the following modifications. Overnight cultures were sub-cultured 1∶25 into fresh BHI and grown to mid-exponential phase (OD_600_ = 0.5–0.6). Each culture was then sub-cultured 1∶100 into BM medium and 200 µl was aliquoted into four replicate wells, followed by incubation at 37°C in a 5% CO_2_ aerobic atmosphere for 48 h. Culture medium was removed by aspiration and wells were gently washed with 200 µl sterile deionized water. Subsequently, 50 µl of a 0.1% solution of crystal violet dissolved in 99% ethanol was applied to each well and incubated at room temperature for 15 min, followed by removal of the fluid by aspiration. Wells were washed twice with 200 µl of water as before and allowed to air dry. The plates were photographed and the wells were de-stained with 200 µl of an acetone:ethanol solution (2∶8) for 30 min at room temperature. The de-staining procedure was repeated and the OD_575_ of the pooled de-staining solution was measured. Results are representative of duplicate assays. Significant differences were determined using Students *t*-test.

### Autolysis Assay

Autolysis was measured as described elsewhere [39], [40], with the following modifications. Overnight cultures were sub-cultured 1∶20 into fresh BHI and grown to late exponential phase (OD_600_ = 0.7). Cells were collected by centrifugation and washed twice in PBS. Cells were resuspended in autolysis buffer (20 mM potassium phosphate buffer, pH 6.5, 1 M KCl, 1 mM CaCl_2_, 0.04% sodium azide) to an OD_600_ of 1.0. The cell suspensions (300 µl) were applied to duplicate wells of a 100-well Bioscreen plate and OD_600_ was monitored at 44°C every 20 min for 10 h in a Bioscreen C lab system. Triplicate cultures of each strain were used.

### Acid Killing Assay

The ability to survive a strong acid challenge was determined as previously described [41], with the following modifications. Briefly, cells from an overnight culture were diluted 1∶25 into BHI broth and incubated to OD_600_ = 0.3 (unadapted) or to OD_600_ = 0.2 followed by a 2-hour incubation period in BHI broth that had been acidified with HCl to pH 5.0 (adapted). Cells were then collected by centrifugation at 3,800×*g* at 4°C, resuspended in 0.1 M glycine buffer, pH 7.4, and vortexed for 1 min. In order to disperse cell clumps, as several strains tended to aggregate, cells were sonicated for two 20-second cycles in a sonicating water bath at room temperature and placed on ice between cycles. Before the start of the assay, aliquots of cells were removed and placed on ice. The remaining cells were then pelleted and resuspended in an equal volume of 0.1 M glycine buffer, pH 2.8, and rotated continuously at room temperature. Duplicate aliquots were removed at 15, 30 and 60 min and diluted 1∶10 in 10 mM Tris-HCL, pH 8.0, and placed on ice. Once the assay was complete, all aliquots were serially diluted in 10 mM Tris-HCL, pH 8.0, and plated on BHI agar followed by a 48 h incubation at 37°C in a 5% CO_2_ atmosphere. Percent survival for each time point was determined by dividing the CFUs of each time point by the initial CFUs multiplied by 100. Data represents the average of two separate experiments performed in duplicate.

### Genetic Competence Assay

Overnight cultures were sub-cultured 1∶20 into fresh BHI and grown to OD_600_ = 0.125. Synthetic competence stimulating peptide (CSP; [36]) was added to final concentrations of 0, 5, 20, 50, 100, or 200 nM. Cells were returned to a 37°C incubator for 15 min before 0.5 µg of the integration vector pBGE [42] was added. Cells were then incubated for 2.5 h and plated on BHI agar plates containing 10 µg ml^−1^ erythromycin. Induction of competence was evaluated after 48 h of incubation at 37°C in a 5% CO_2_ atmosphere. Competence induction was determined by comparing the number of resulting colonies of a particular strain as a function of the concentration of input CSP.

### Western Blots

Cells from mid-exponential phase cultures (OD_600_ = 0.5) grown in BHI, were collected by centrifugation at 3,800×*g* for 10 min. The culture supernates were filtered through a 0.2 µm syringe filter and proteins from a 700 µl aliquot (standardized by OD_600_) were precipitated with an equal volume of 20% TCA overnight at −20°C. Precipitated proteins were washed once with 300 µl cold acetone and allowed to air dry before the pellets were resuspended in 50 µl TE (50 mM Tris-HCL, 1 mM EDTA, pH 7.5). The suspension was combined with 50 µl 2X SDS-PAGE sample buffer, boiled for 5 min and centrifuged at top speed for 3 min in a microcentrifuge. Cell-wall associated proteins were extracted by boiling cell pellets (adjusted to the same OD_600_) in 1X SDS-PAGE sample buffer for 5 min, followed by centrifugation to remove whole cells. Proteins from 25 µl of the culture supernates and cell-wall extracts were separated on 4–8% XT Criterion Tris-acetate gradient gels. Proteins were transferred to nitrocellulose for Colloidal Gold Total Protein Stain (Bio-Rad) or PVDF membranes for Western blotting with a rabbit anti-GtfB/C polyclonal antisera (a kind gift from W. H. Bowen, University of Rochester [43]). Western blots were reacted with a 1∶500 dilution of the antisera and developed according to the supplier’s directions using the Amersham ECL Western blot kit.

### *Galleria* Mellonella Virulence Assay

Stationary phase (16 h) *S. mutans* cultures grown in BHI at 37°C in 5% CO_2_ were diluted 1∶20 into fresh BHI supplemented with 5% horse serum. Cultures were grown to OD_600_ = 0.6 and placed on ice for at least 30 minutes. Cultures were washed twice with an equal volume of sterile saline solution (0.9% NaCl) and adjusted to approximately 5×10^7^ CFU/ml in sterile saline. Bacterial colony counts on trypticase soy agar (TSA) plates were used to confirm initial inocula. A negative control for infection was prepared using heat-killed OMZ175 (15 min at 75°C). *G. mellonella* larvae in the 4th–5th instar stages, sorted by weight (200 to 300 mg) and showing no signs of melanization were randomly chosen and kept at 4°C prior to injection. A 10-µl Hamilton syringe was used to inject 5-µl aliquots of bacterial inoculum into the hemocoel of each larva via the last left proleg. After injection, larvae were kept at 37°C under atmospheric conditions and survival was recorded at selected intervals. Kaplan-Meier killing curves were plotted and estimation of differences in survival were compared using the log-rank test. A *P* value ≤ 0.05 was considered significant. All data was analyzed with GraphPad Prism 5.0 software. In addition to *cnm*+ and *cnm*- control strains (OMZ175 and UA159, respectively), a *cnm*-inactivated mutant strain, OMZ175*-cnm*, was also included to serve as a control to monitor Cnm dependent larvae death [10].

### PCR to Confirm Presence or Absence of Genes

PCR was used to confirm the absence of intact *comC*, *comD* and *comE* genes in Smu56 and Smu57. The following primers were used in a standard PCR using colonies of UA159 (positive control), Smu56 or Smu57 as template; *comD*, SP01F-2 (5′-GAATGAAGCCTTAATGATACTTT-3′) and SP01R (5′CTATTTTATTATTAGGAGTTGCTTGAATA 3′), *comE*, SP02F (5′ –ATGATTTCTATTTTTGTATTGGAAGAT-3′) and SP02R (5′-TCATTTTGCTCTCCTTTGATCAGCAAT-3′), *comC*, SP03F (5′-GGAGTATAAAATGAAAAAAACACTA-3′) and SP03R (5′-GCC-TATCTTATTTTCCCAAAGCTT-3′). To confirm the presence or absence of intact Smu.1008 and Smu.1009 in Smu81, primers SP26F (5′-GTAGAATAAAGTTATGCTAAAGCAA-3′) and SP26R (5′-GATTACGCAACAATCATAGCTGTTT-3′), were used in a standard colony PCR reaction.

## Results

### Selection of Sequenced Strains for Further Characterization

The strains utilized in this study are described in Table 1. To better understand the scope of possible phenotypes within the species *S. mutans*, strains from two geographically-diverse collections of clinical isolates [30], [44], [45] were sampled based on genetic diversity determined by WGS sequences [34]. Figure S1 depicts gene content differences between strains based on orthologs recovered across genomes via an all-versus-all BLASTP search combined with clustering using OrthoMCLS [46]. Strains were selected for further phenotypic characterization based on their clustering, the presence or absence of selected non-core genes, and preliminary observations of phenotypic behaviors.

Through genome sequence analysis we were surprised to discover that one of the isolates characterized in this study, Smu77/NV1996 [30], was actually a genetically-engineered derivative of *S. mutans* V403 containing insertionally-inactivated *gtfBC*, *gtfD* and *ftf* genes, and thus represents strain V1996 originally described by Munro *et al.* [47]. It has been confirmed that Smu77 is resistant to kanamycin, erythromycin and tetracycline and contains the *aphA* gene interrupting *gtfB-gtfC* [47], the *tetM* gene within the *gtfD* gene [48], and *ermAB* inserted within the gene for *ftf* [49]. V403 is a serotype *c* strain isolated from human blood at the CDC in Atlanta, GA and contains a 5.6 kb cryptic plasmid (pVA403) [50]. V403 has also been reported to contain the *cnm* gene and is able to bind type-1 collagen [51].

### Stress Tolerance Varies Widely among Genetically-diverse Isolates

Growth curve analysis was performed for the 15 strains under various stress conditions and compared to the well-characterized reference strain UA159. Most strains grew at similar rates and to similar final optical densities under non-stressed conditions (BHI, pH 7.5, with an oil overlay) (Table 2), but Smu56 had an unusually long doubling time (129±4 min) under non-stressed conditions. Although all experiments were conducted with fresh isolates from freezer stocks, we noted that Smu56 was viable on agar plates stored at 4°C for only 2 to 3 days, whereas most of the other isolates remained viable for much longer.

**Table 2:** Growth characteristics of clinical isolates in different stress conditions.

Growth condition(BHI)		Smu 159	Smu 20	Smu 21	Smu 44	Smu 52	Smu 56	Smu 57	Smu 63	Smu 69	Smu 77	Smu 81	Smu 86	Smu 93	Smu 98	Smu 104	Smu 109
T_d_ range(min.)																	
pH 7.5	T_d_	**65±2**	71±0^€^	73±3	69±1^€^	94±3*	**129±4** *	78±1*	80±2*	114±1*	106±1*	84±3*	70±1^€^	75±2*	72±1^€^	90±2*	77±2*
65 to 129	Lag	100	100	160	100	180	160	160	100	120	140	120	100	100	160	100	120
	OD_m_	0.74	0.73	0.65	0.74	0.59	0.64	0.60	0.72	0.63	0.65	0.76	0.73	0.72	0.67	0.70	0.70
	OD_f24_	0.60	0.59	0.54	0.61	0.52	0.59	0.51	0.56	0.48	0.55	0.62	0.63	0.62	0.53	0.61	0.59
pH 5.5	T_d_	172±5	176±3	164±5	**157±7** †	211±6*	264±4*	237±4*	226±13*	233±8*	259±9*	255±11*	**328±1** *	201±6*	274±50^€^	214±8*	222±6*
157 to 328	Lag	180	200	220	180	200	300	280	260	200	200	220	200	160	200	180	160
	OD_m_	0.60	0.61	0.43	0.53	0.48	0.25	0.37	0.54	0.48	0.61	0.55	0.58	0.53	0.39	0.58	0.55
	OD_f24_	0.52	0.51	0.40	0.43	0.44	0.23	0.36	0.52	0.43	0.56	0.49	0.51	0.50	0.38	0.48	0.53
O_2_ Air	T_d_	87±3	107±14†	94±3†	88±1	134±45	**218±10** *	127±34	93±2†	162±10*	128±7*	113±5*	89±3	**86±2**	88±5	108±15†	86±6
86 to 218	Lag	220	180	160	120	160	340	200	100	200	280	120	100	100	160	180	160
	OD_m_	0.54	0.50	0.57	0.61	0.48	0.51	0.46	0.57	0.46	0.50	0.63	0.65	0.67	0.64	0.57	0.67
	OD_f15_	0.41	0.26	0.25	0.53	0.22	0.49	0.36	0.46	0.31	0.32	0.41	0.49	0.36	0.39	0.29	0.35
	OD_f45_	0.29	0.34	0.20	0.52	0.30	0.46	0.34	0.22	0.22	0.21	0.61	0.57	0.44	0.33	0.62	0.54
Paraquat	T_d_	129±9	172±6*	107±3^€^	149±3^€^	**387±15** *	173±14	111±8†	131±4	202±5*	206±16*	**NG**	**105±0** ^€^	117±3†	122±11	142±0†	137±18
105 to 387	Lag	660	660	520	520	700	700	600	400	480	1000	NA	460	500	800	520	540
	OD_m_	0.52	0.54	0.63	0.56	0.37	0.61	0.59	0.57	0.47	0.57	0.14	0.67	0.70	0.61	0.74	0.67
	OD_f48_	0.49	0.52	0.53	0.52	0.37	0.54	0.55	0.54	0.42	0.55	0.09	0.61	0.67	0.60	0.70	0.60
CSP	T_d_	100±0	85±3*	83±3*	**68±2** *	98±2	137±2*	78±6*	95±3†	104±3†	133±4*	87±10†	**240±7** *	73±1*	91±1*	112±6^€^	89±4^€^
68 to 240	Lag	200	180	200	180	220	260	240	160	180	200	120	440	140	180	200	200
	OD_m_	0.60	0.63	0.58	0.71	0.58	0.58	0.52	0.52	0.56	0.53	0.71	0.29	0.66	0.64	0.65	0.64
	OD_f20_	0.46	0.46	0.43	0.59	0.46	0.50	0.44	0.39	0.45	0.46	0.58	0.28	0.58	0.54	0.56	0.52

Mean exponential growth rate (Td) is expressed in minutes and was determined based on growth curves generated in a Bioscreen C Lab System. Lag time is expressed as minutes. OD_f_ indicates the average final OD at the indicated time in hours. OD_m_ indicates the average maximum yield (highest OD reached during growth). Growth curve data is based on duplicate growth curves of two separate cultures (n = 4). Bold values represent the highest and lowest Td under each condition. Statistical differences compared to reference strain UA159 are indicated;

* = *P*≤0.001,

€ = *P*≤0.01, and

† = *P*≤0.05.

Aciduricity, the ability to grow and to continue to produce acids at low pH, is an important virulence attribute for *S. mutans*, and growth rate and final yield at pH 5.5 are considered to be good measures of aciduricity [52]. When strains were cultured in medium that had been acidified to pH 5.5 with HCl, substantial variation in growth characteristics were observed, with mean doubling times ranging from 157 to 328 minutes and maximum yields ranging from OD_600_ values of 0.25 to 0.61. Strain Smu44 grew at a significantly faster rate at pH 5.5 than strain UA159 (Td 157±7 compared to 172±5, *P*≤0.05). Smu21 also displayed consistently faster exponential growth than UA159, although the difference was not significant (164±5, *P*≤0.09). Despite the faster exponential growth of Smu21 and Smu44 at pH 5.5, the cell yields were less than for UA159 (OD_600 max_ 0.43 and 0.53, respectively, compared to 0.60 for UA159).

**Figure 1:**
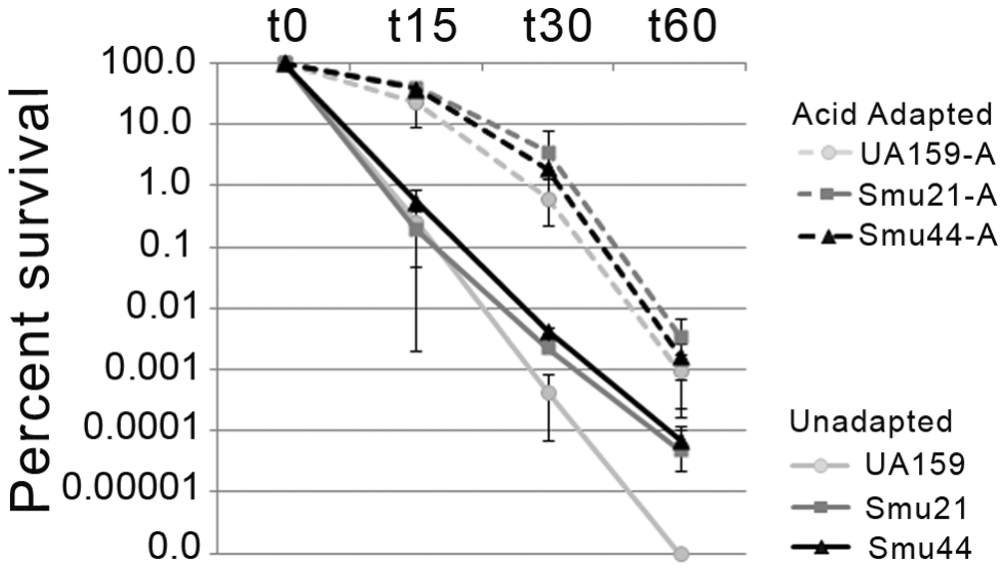
Acid killing - Percent survival after exposure to pH 2.8. Cells were grown to mid-exponential phase, washed in 0.1 M glycine buffer pH 7.4 and re-suspended in 0.1 M glycine pH 2.8. Cells were removed at 15-, 30- and 60-minute intervals, serial diluted and plated. For acid adaptation, cells were harvested at early exponential phase and re-suspended in pH 5.0 media and grown for 2 additional hours. Percent survival was determined based on the number of cells at each time point divided by the number of cells before acid challenge, t0. Data represents the average of two separate experiments performed in duplicate.

The ability of Smu21 and Smu44 to survive a low pH challenge (pH 2.8) before and after acid adaptation at pH 5.0 for 2 hours was also compared (Figure 1). Both strains exhibited better survival over time at low pH compared to UA159 when cells had not been previously acid-adapted. In contrast, all strains performed similarly when they were first allowed to adapt to growth at low pH. This finding supports the idea that Smu21 and Smu44 have a greater constitutional resistance to acid stress than UA159, consistent with the growth curve data. When the ATPase activity of un-adapted and acid-adapted cells was compared between these strains, greater ATPase activity was consistently observed in acid-adapted cells compared to un-adapted (data not shown). However, when ATPase activity was compared between these strains no significant differences were seen under the conditions tested.

Oxidative stress tolerance is a critically important factor in the establishment, persistence and ecology of oral bacteria, and thus affects the pathogenic potential of oral biofilms in major ways [53], [54]. Organisms in the oral cavity are transiently exposed to different oxygen levels, and to different types and quantities of reactive oxygen species (ROS) generated in saliva and within oral biofilms [53], [54]. Sensitivity to oxygen was determined by examining growth in a Bioscreen C machine in the presence of air (no mineral oil overlay), revealing major differences in doubling times (86 to 218 min), with cell yields ranging from OD_600_ values of 0.46 to 0.67 (Table 2, O_2_ Air OD_max_). When some strains (Smu81, Smu86, Smu104 and Smu109) reached stationary phase, a sharp drop in OD_600_ values occurred; followed by a resumption of growth with cell yields reaching similar values to the maximum yield at 48 hours (Figure S2). For example, strain Smu104 reached a maximum cell yield of 0.57 after about 6 hours of growth, the OD of the culture then declined to 0.29 after 15 hours, followed by regrowth of the culture to attain a final yield of OD_600_ = 0.62 at 48 h. Multiple other strains did not show this growth-lysis-regrowth cycle, displaying more typical plateaus in stationary phase, or a slow and steady decline in optical density during stationary phase.

Similar to cells exposed to air, growth in 25 mM paraquat, which can generate superoxide anion, yielded great variation in growth rates and final yields, with one strain (Smu81) unable to initiate growth in medium containing paraquat (Table 2). Unlike for cells growing in the presence of air, however, evidence of the stationary-phase lysis, or lysis and regrowth, was not observed in cells cultured in paraquat. It should also be noted that none of the strains were able to grow in the presence of paraquat unless the wells were overlaid with mineral oil.

### Autolysis Varied among Clinical Isolates

**Figure 2:**
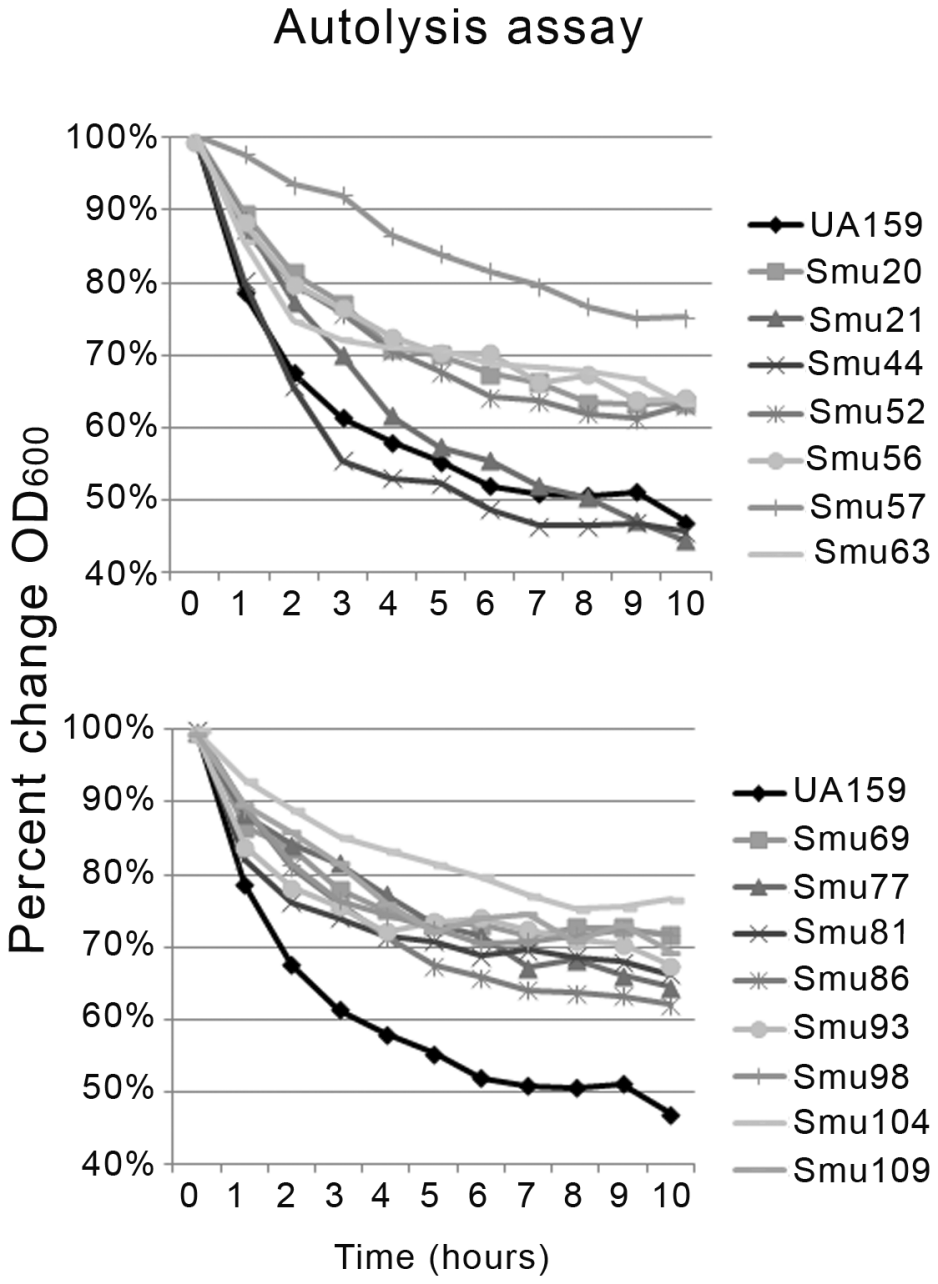
Autolysis assay. Late exponential phase cells were suspended in autolysis buffer and OD_600_ was monitored over 10 hours. Results are the average of triplicate assays and are represented as percent change in OD_600_ over time.

Autolysis is a natural process whereby cells undergo lysis in response to an environmental signal; this process is important for biofilm formation, competence development and cell wall turnover [55]. In *S. mutans*, the peptidoglycan hydrolase AltA [40] has been shown to be a significant contributor to autolysis [56]. Autolysis has also been shown to stimulate eDNA accumulation, which can enhance biofilm formation and alter biofilm architecture [57]. In order to examine autolytic behavior within the sequenced isolates, the change in OD_600_ over time of late-exponential phase cells that were washed, resuspended in autolysis buffer, and incubated at 44°C was monitored (Figure 2). Smu44 was the most autolytic strain, displaying slightly more lysis than UA159, whereas Smu21 showed slightly less autolysis than UA159. Smu57 and Smu104 were the least autolytic of strains tested, with the remaining strains displaying an intermediate level of autolysis. All strains contained the *altA* gene, and in most cases the gene sequence was identical to that of UA159. There was no correlation between the autolysis phenotype of strains observed here and the results of growth experiments in the presence of oxygen described above.

### Competence Development Correlates with Sensitivity to CSP and the Presence of Bacteriocins and Immunity Proteins

Much attention has been focused recently on the role of peptide-based quorum sensing systems that control bacteriocin production and genetic competence, as well as cell lysis and altruistic cell death. One intensively-studied peptide in *S. mutans* is competence stimulating peptide (CSP), which is sensed by the ComDE two-component system to activate bacteriocin production and DNA uptake [58]. Low concentrations of CSP (e.g. 30 to 100 nM) are sufficient to stimulate competence [59], whereas high concentrations of CSP (2 µM) can induce death in sub-populations of *S. mutans* [60], in part due to production of an endogenously-acting bacteriocin, mutacin V (CipB). There is also evidence that the expression of a number of bacteriocins (NlmD, Smu.1906 and CipB) can affect the level of induction of genetic competence through the CSP pathway [61]. To examine the sensitivity to CSP of the various isolates, cells were grown in BHI broth containing 400 nM synthetic CSP. There were some striking differences among the clinical isolates with respect to CSP sensitivity, with certain strains showing no adverse effects when CSP was present (Smu44, Smu56, Smu57, Smu81, and Smu93). With the exception of Smu93, these resistant strains could not be made naturally competent for genetic transformation, even with addition of exogenous CSP. In fact, out of the 15 strains tested, we were unable to obtain transformants in seven isolates when cells were cultured in the presence of 200 nM CSP in BHI medium. In four of these seven strains (Smu56, Smu57, Smu81, Smu104; Table 3), the lack of CSP induced competence could be potentially attributed to the absence of one or more Com-related proteins. Specifically, in Smu56 and Smu57, *comCDE* are absent (not present in the draft genome and not detectable by PCR), whereas *comE* is present but truncated in Smu81 (Figure S3). Also in Smu81, *comC* contains a frameshift that introduces a nonsense mutation (Figure S4) and ComX is truncated in Smu104 due to a nonsense mutation 127-bp into the *com*X gene (Figure S5). Notably, Smu56, Smu57, Smu81 and Smu104 contained ComRS, but none of these strains yielded transformants when grown in the presence of 600 nM XIP in a chemically-defined medium (data not shown).

**Table 3:** Mean generation time in BHI with CSP, presence of genetic competence, *com* genes and bacteriocin distribution among clinical isolates.

	Smu 159	Smu 20	Smu 21	Smu 44	Smu 52	Smu 56	Smu 57	Smu 63	Smu 69	Smu 77	Smu 81	Smu 86	Smu 93	Smu 98	Smu 104	Smu 109
T_d_ CSP	100±0	85±3	83±3	68±2*	98±2*	137±2	78±6*	95±3	104±3^†^	133±4	87±10*	240±7	73±1*	91±1	112±6	89±4
Competence	Y	Y	Y	N	Y	N	N	Y	N	Y	N	Y	Y	N	N	Y
*com* genes		*comC*				*comC* *comDE*	*comC* *comDE*		*comCΔ*		*comEC*		*comCΔ*		*comX*	
*Smu.1902*	+	−	+	−	+	−	−	−	+	+					+	−
*Smu.1906*	+	+	+	+	+			+	+	+^a^	+^a^	+	+	+	+	+
*nlmA/B*	+/+	+/+	+/−	+/−	+/+	+/−	−/−	+/+	−/−	+/+	−/−	+/+	+/+	−/−	+/+	−/−
*nlmD*	+	+	+^b^	+	+	+^b^	+b c	+	+	+	+	+^d^	+	+	+^d^	+^b^
Smu.1914/*cipB*	+	+			+			+	+	+^a^	+^a^	+	+	−	+	+
Smu.1909	+			+	+			+	+			+	+	−	+	+
Smu.1913	+	?			+			+	+			+	+	−	+	+
Smu.925/cipI	+	+e f	+^g^	+	+	+^f^	+	+^f^	+	+e f	+^e^	−	+^e^	−	+e f	−
Smu.152	+	+	+	+	+	+	−	+	−	+	−	+	+	−	+	−

Mean generation times (Td) are same as in Table 1 (CSP). An * indicates Td is not significantly different from Td in BHI without CSP. The ^†^ for strains Smu69 indicates Td in CSP was significantly better (P≤0.003) with CSP compared to without CSP. Absence of *com* genes was determined by whole genome sequence analysis and confirmed by PCR for Smu56 and Smu57. ComC from Smu69 has a truncated comC gene, which is missing the last 3 C-terminal amino acids. For competence Y = yes and N = no. Putative bacteriocins: *nlmA/B* = Smu.150/Smu.151, *nlmD* = Smu.423, *cipB* = Smu.1914, Smu.1906. Putative immunity proteins: Smu.1909, Smu.1913, *cipI* = Smu.925, Smu.152. (Smu.1906),

a = there appears to be a recombination between Smu.1914 and Smu.1906 resulting in a hybrid protein that contains the signal sequence of Smu.1914 and the bacteriocin sequence from Smu.1906. (NlmD),

b = mutated signal sequence,

c = truncated by 12 aa,

d = insertion of two nucleotides resulting in a frameshift at amino acid 50. (Smu.925/CipI),

e = ORF starts downstream at 54 bps ATG or GTG site,

f = a frame-shift mutation results in a premature stop codon leading to a truncation of 4 aa at C-terminus,

g = truncated by 7 aa at C-terminus due to a frame-shift mutation.

In strains Smu69 and Smu93, ComC is predicted to be truncated by 3 amino acids because of an insertion of 18 nucleotides within the *comC* gene (Figure S6). Surprisingly, Smu69 had a faster generation time when growing in the presence of CSP (104±3 min) than the absence of CSP (114±1 min), while Smu93 was unaffected by the presence of CSP (Td 75±2 v.s. Td 73±1). The distribution of predicted bacteriocins (Smu.1902, Smu.1906, Smu.150, Smu.151, Smu.423 and Smu.1914) and putative immunity proteins (Smu.1909, Smu.1913, Smu.925, and Smu.152) also varied among strains (Table 3). In general, we noted that strains that were not able to become competent for natural transformation contained the genes for fewer bacteriocin and immunity proteins than strains that were competent for natural transformation.

**Figure 3:**
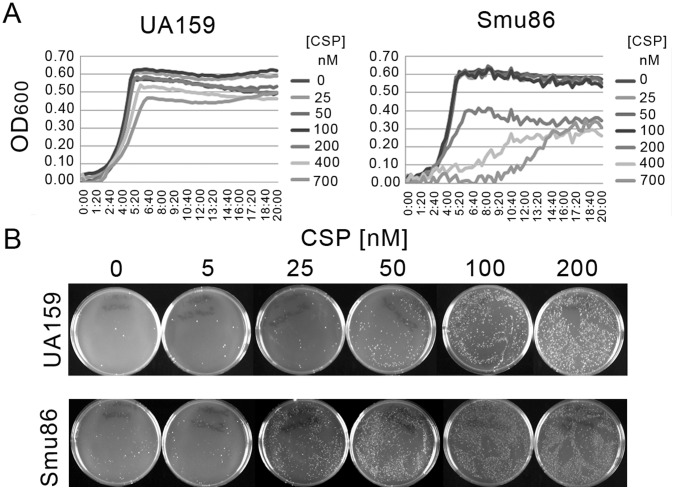
Sensitivity to CSP. Growth sensitivity to CSP correlates with level of competence. A) Growth curves of UA159 and Smu86 in various concentrations of CSP. B) Transformation assay with various concentrations of CSP. The photograph of the plates is representative of duplicate biological replicates.

Strain Smu86 was the most sensitive to growth inhibition by CSP, with the greatest lag and longest doubling time of all the strains tested (7 h lag, 240 min Td). To test whether the increased sensitivity to growth inhibition by CSP was correlated with competence development, cells were cultured in various concentrations of CSP (25–700 nM) and growth was compared with the reference strain UA159 under the same conditions (Figure 3A). Competence induction was also measured using various concentrations of CSP (0–200 nM; Figure 3B). The growth of Smu86 was inhibited to a greater extent at lower concentrations of CSP than UA159, such that 700 nM CSP was required to elicit the same degree of growth inhibition in UA159 as was seen with 200 nM CSP in Smu86. A similar effect was seen with competence development, where only one-fourth the amount of CSP was needed to stimulate an increase in transformation in Smu86 (25 nM) as that required for UA159 (100 nM).

### Biofilm-forming Capacity Varied Widely among Strains

*S. mutans* encodes three functionally-distinct glucosyltransferase enzymes, GtfB, GtfC, and GtfD, that contribute to various degrees to sucrose-dependent adhesion [11]. GtfD is found primarily in cell-free extracts and forms soluble glucan polymers dominated by α1,6- linked glucose chains, whereas GtfB is primarily cell-associated and catalyzes the synthesis of insoluble glucans composed mainly of α1,3 linkages [62]. GtfC can form both soluble and insoluble glucans and is found cell-associated and in culture supernates. *S. mutans* also encodes four glucan binding proteins, GbpA, GbpB, GbpC, and GbpD, that impact biofilm formation, as reviewed in [63].

**Figure 4:**
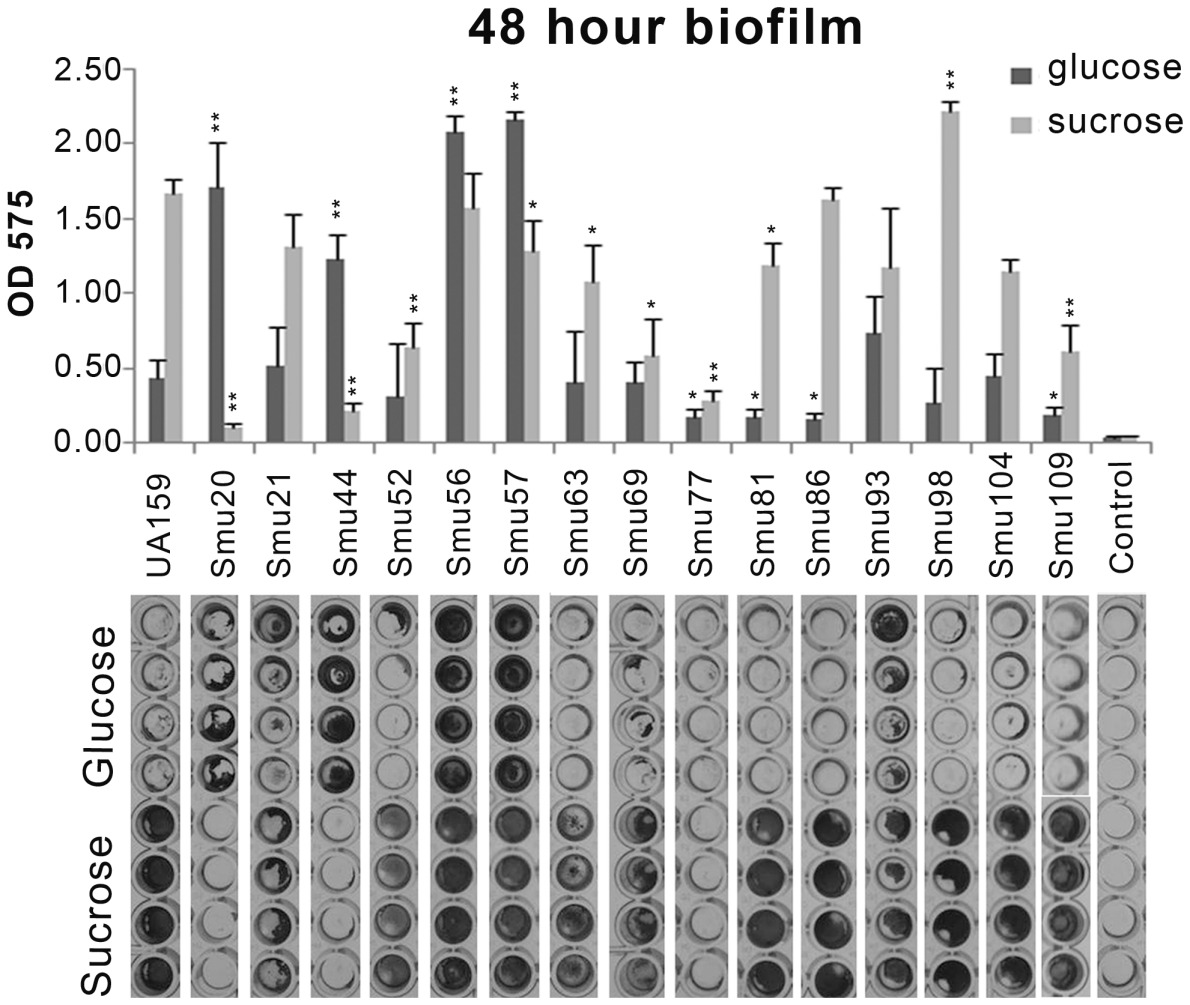
Biofilm formation by clinical isolates in semi-defined media with 20 mM glucose or sucrose. Top panel shows numeric values of crystal violet biofilm assay (OD_575_ of eluted solution). Statistically significant differences compared to UA159 are indicated, ** = *P*≤0.001, * = *P*≤0.05. Bottom panel shows a picture of the crystal violet stained biofilm for each strain in the micro-titer plate wells before de-stain procedure.

**Figure 5:**
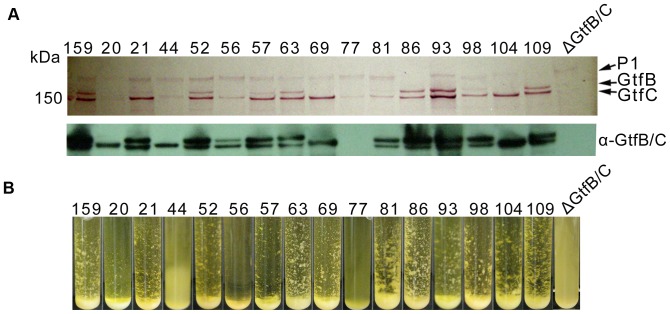
Expression of cell-wall associated proteins involved in biofilm formation and sucrose mediated cell clumping. A) Cell associated protein extracts were from cell pellets harvested from mid-log BHI cultures. Proteins were separated on a 4–8% XT Criterion (BioRad) tris-acetate gel, transferred to a nitrocellulose membrane and stained with BioRad Colloidal Gold Total Protein Stain. For Western blot, proteins were transferred to PVDF membranes and reacted with polyclonal antisera that recognizes both GtfB and GtfC. B) Photos were taken after 48 hours of growth in BHI supplemented with 0.3% sucrose.

*In vitro* biofilm development was analyzed after 48 hours of growth in a semi-defined medium containing 20 mM glucose or 20 mM sucrose (Figure 4). As has usually been noted for *S. mutans*, most strains formed more robust biofilms in sucrose than in glucose. Surprisingly, Smu20 and Smu44 formed more biofilm in glucose than sucrose, and some strains formed biofilms to a similar degree in sucrose and glucose (Smu56 and Smu57). The *gtfBCD* mutant strain Smu77/V1996 of V403 formed almost no biofilm in either of the tested conditions. The *gtfB* and *gtfC* genes are co-transcribed and share a high degree of similarity, so we could not unequivocally ascertain the complete content or organization of these genes in the contigs of all draft genomes. Therefore, to more reliably correlate the results of the biofilm assay with the distribution of Gtf enzymes within the clinical isolates, cell-associated and culture supernatant proteins from mid-exponential phase (BHI) cultures were separated by SDS-PAGE and analyzed by Western blotting using a polyclonal antisera that recognizes both GtfB and GtfC [43]. The results (Figure 5) revealed differences between strains, with 10 strains clearly expressing different amounts of both GtfB and GtfC and 2 strains only expressing one band corresponding to GtfC (Smu69, Smu104). Samples prepared from Smu20 and Smu44 also contained only one band that was recognized by the GtfB/C polyclonal antibody and migrated between GtfB and GtfC, indicating the likelihood that a recombination event between *gtfB* and *gtfC* occurred in these strains [64]–[66]. Such a recombination event may account for the poor biofilm formation by these strains in BM-sucrose medium. Mutant strain Smu77/V1996 is lacking any cell-associated GtfB or GtfC protein (Figure 5A; bottom panel) consistent with the mutations described in [47] within the *gtfB-gtfC* genes. A Δ*gtfBC* mutant of UA159 that was constructed and verified in our laboratory (SAB109) [67], was used as a negative control for the Western blot.

Because it is possible for a polyclonal antibody to recognize a non-functional protein, we examined the ability of cells to aggregate in the presence of sucrose in broth cultures (Figure 5), a characteristic that requires GTF activity. Strains Smu20, Smu44, Smu77/V1996 and a Δ*gtfBC* mutant (SAB109) showed very little sucrose-dependent aggregation (Figure 5), consistent with the biofilm results and the Western blots showing aberrant Gtf production. On the other hand Smu56 and Smu57, both of which formed robust biofilms in both sucrose and glucose, displayed somewhat different phenotypes in this assay. Smu56 did not form aggregates or stick to the sides of glass test tubes, as was seen with other strains, and instead settled to the bottom of the tube. Smu57 formed cell aggregates more similar to the other Gtf-positive strains (Figure 4). Based on the genome sequences, all strains contained the *gtfD* gene sequence and all except Smu77/V1996 expressed a band at ∼160 kDa in culture supernatant preparations corresponding to the known size of GtfD (data not shown). On further examination, Smu77/V1996 contained a truncated form of *gtfD,* which is the result of an insertion into the *Bgl*II site of *gtfD* of a 4605-bp of sequence containing ORFs 11–14 of transposon Tn916 from *Enterococcus faecalis* DS16 (Figure S7). This is slightly different from what was described in [48] for V1996, where the *gtfD* gene was interrupted with a 5.4-kb *Bam*HI digest from pLN2 [68] that contained the *tetM* gene from Tn916.

A comparison of the gene content of the clinical isolates revealed there were differences in other genes that could affect the ability to form biofilm. For example Smu77/V1996 (V403, Δ*gtfBCD*, Δ*ftf*), Smu81, Smu86, Smu98 and Smu109 appear to be missing the gene for the major glucan binding protein, GbpA. With the exception of Smu98, it is noteworthy that these same strains also contain the *cnm* gene that encodes a protein with a collagen binding domain and an LPXTG motif at its C-terminus for cell-wall anchoring [51], which will be discussed in greater detail below. Furthermore, Smu86 and Smu109 also appear to express less of the cell-surface saliva binding adhesin P1 [69] compared to other strains (Figure 5A, top panel).

### Distribution of Two-component Signal Transduction Systems in Draft Genomes of Isolates

The genome of *S. mutans* UA159 revealed 13 putative two-component signal transduction systems (TCS) and one orphan response regulator (GcrR) [25]. Recently, a 14^th^ TCS (Smu.45/Smu.46) was described in UA159, but this TCS could only be found in 2 of the 13 strains analyzed [70]. Apparent Smu.45/Smu.46 homologs were found in 12 of the 57 strains sequenced here, but were in only one (Smu52) of the 15 isolates analyzed in this study. Biswas *et al*., 2008 also noted that the histidine kinase (HK) of TCS-5 (Smu.1814) was only found in two of the 13 strains examined in that study. We found that TCS-5 (Smu.1814/Smu.1815) was present in 45% of the strains sequenced and was not identified in 11 of the 15 strains characterized here (Table 4). Another recent study detailed the distribution of 18 TCSs found in eight newly-sequenced mutans streptococci (*S. sobrinus* DSM20742, *S. ratti* DSM20564 and 6 *S. mutans* strains) [71]. Song identified a 15^th^ TCS (ComP/CmpR) in one *S. mutans* serotype *f* strain isolated from blood [71]. The sensor HK protein is predicted to contain 10 transmembrane domains, and is classified as a membrane-sensing HK and can be further classified into subgroup (ii) as a quorum-sensing HK with ComD (TCS-13). The response regulator (RR) of this novel HK/RR pair contains a LuxR_C-like DNA-binding HTH domain and was classified as a NarL type RR, which in other bacteria are involved in the control of genes that affect nitrogen fixation, sugar phosphate transport, nitrate and nitrite metabolism, quorum sensing or osmotic stress tolerance [72]. The function of this TCS in *S. mutans* has yet to be determined. However, a recently sequenced serotype *k* strain (LJ23) also contains TCS-15 [73]. In our analysis of the genomes of the 57 isolates from across the globe, TCS-15 was found in 16 of the 57 strains, and was distributed across all serotypes. Six of the strains phenotypically characterized in this study (Smu21, Smu77/V1996-V403, Smu81, Smu86, Smu98, Smu109) have TCS-15 (Table 4). While most strains contain TCS-7 (Smu.1037/Smu.1038) of unknown function, Smu81 contained several mutations resulting in frameshifts in both the HK and RR of TCS-7, whereas Smu109 has frameshift mutations in only the RR (Smu.1038). Furthermore, according to the draft genome sequence of Smu81, it appears this strain is also missing TCS-8 (Smu.1009/Smu.1008) [74], however these genes were able to be amplified by PCR using gene specific primers, indicating there is a gap in the sequence for this strain and therefore the presence of mutations within this TCS can not be ruled out. Smu56 and Smu57 are completely missing *comDE* (TCS-13), which are important for genetic competence, biofilm formation, bacteriocin production, quorum sensing, and stress tolerance [56], [75]–[77].

**Table 4:** Absence of two-component systems in clinical isolates.

TCS HK/RR	Smu 159	Smu 20	Smu 21	Smu 44	Smu 52	Smu 56	Smu 57	Smu 63	Smu 69	Smu 77	Smu 81	Smu 86	Smu 93	Smu 98	Smu 104	Smu 109
TCS-1 (Smu.1516/.1517)	+/+	+/+	+/+	+/+	+/+	+/+	+/+	+/+	+/+	+/+	+/+	+/+	+/+	+/+	+/+	+/+
TCS-2 (Smu.1128/.1129)	+/+	+/+	+/+	+/+	+/+	+/+	+/+	+/+	+/+	+/+	+/+	+/+	+/+	+/+	+/+	+/+
TCS-3 (Smu.1145c/.1146c)	+/+	+/+	+/+	+/+	+/+	+/+	+/+	+/+	+/+	+/+	+/+	+/+	+/+	+/+	+/+	+/+
TCS-4 (Smu.928/.927)	+/+	+/+	+/+	+/+	+/+	+/+	+/+	+/+	+/+	+/+	+/+	+/+	+/+	+/+	+/+	+/+
TCS-5 (Smu.1814/.1815)	+/+	−/−	−/−	+/+	+/+	−/−	−/−	−/−	+/+	−/−	−/−	+/+	−/−	−/−	−/−	−/−
TCS-6 (Smu.660/.659)	+/+	+/+	+/+	+/+	+/+	+/+	+/+	+/+	+/+	+/+	+/+	+/+	+/+	+/+	+/+	+/+
TCS-7 (Smu.1037c/.1038c)	+/+	+/+	+/+	+/+	+/+	+/+	+/+	+/+	+/+	+/+	−/−	+/+	+/+	+/+	+/+	+/−
TCS-8 (Smu.1009/.1008)	+/+	+/+	+/+	+/+	+/+	+/+	+/+	+/+	+/+	+/+	+/+*	+/+	+/+	+/+	+/+	+/+
TCS-9 (Smu.1965c/.1964c)	+/+	+/+	+/+	+/+	+/+	+/+	+/+	+/+	+/+	+/+	+/+	+/+	+/+	+/+	+/+	+/+
TCS-10 (Smu.577/.576)	+/+	+/+	+/+	+/+	+/+	+/+	+/+	+/+	+/+	+/+	+/+	+/+	+/+	+/+	+/+	+/+
TCS-11 (Smu.486/.487)	+/+	+/+	+/+	+/+	+/+	+/+	+/+	+/+	+/+	+/+	+/+	+/+	+/+	+/+	+/+	+/+
TCS-12 (Smu.1548c/.1547c)	+/+	+/+	+/+	+/+	+/+	+/+	+/+	+/+	+/+	+/+	+/+	+/+	+/+	+/+	+/+	+/+
TCS-13 (Smu.1916/.1917)	+/+	+/+	+/+	+/+	+/+	−/−	−/−	+/+	+/+	+/+	+/−	+/+	+/+	+/+	+/+	+/+
TCS-14 (Smu.45/.46)	+/+	−/−	−/−	−/−	+/+	−/−	−/−	−/−	−/−	−/−	−/−	−/−	−/−	−/−	−/−	−/−
TCS-15 (CmpR/ComP)	−/−	−/−	+/+	−/−	−/−	−/−	−/−	−/−	−/−	+/+	+/+	+/+	−/−	+/+	−/−	+/+
Orphan RR (Smu.1924)	+	+	+	+	+	+	+	+	+	+	+	+	+	+	+	+

Presence or absence of genes are indicated as a + or − sign. TCS-1 = VicKR, TCS-2 = CiaHR, TCS-3 = CovSR, TCS-4 = KinF/LlrF, TCS-5 = ScnKR, TCS-6 = SpaKR, TCS-7 = PhoR/YcbL, TCS-8 = KinG/LlrG, TCS-9 = LevSR, TCS-10 = LysST, TCS-11 = LiaSR, TCS-12 = HK11/RR11, TCS-13 = ComDE, Orphan RR = GcrR.

* = presence of genes Smu.1008-Smu.1009 was confirmed by PCR, but gene sequence is unknown for these proteins in Smu81, so point mutations could not be ruled out.

HK- sensor histidine kinase, RR- response regulator.

### Identification of Novel Genes and Assessment of their Contribution to Virulence in *Galleria Mellonella*

The Type VII secretion system, found exclusively in Gram-positive bacteria (reviewed in [78], [79]), is responsible for the secretion of the WXG100 family of effector proteins, ESAT-6/EsxA and CSP-10/EsxB, which are required for the virulence of *Mycobacterium tuberculosis* [79] and necessary for persistent infection of *Staphylococcus aureus* [80]. Components of a Type VII secretion system (T7SS) were identified in 8 of the 57 *S. mutans* strains (see Table S1). These strains contained apparent homologs of EsxA, EsaA, EssA, EsaB, EssB and EssC (FtsK-SpoIIIE-domain), of which EssA, EssB and EssC are minimally required for secretion of EsxA or EsxB in *S. aureus* [80]. There was no EsxB homolog detected in any of the *S. mutans* strains, however there is at least one, and as many as four, EsaC-like proteins in those strains that encode a Type VII secretion (See Figure S8 and Table S1). The EsaC protein of *S. aureus* is a 130-aa soluble effector protein of the T7SS, which is negatively regulated post-transcriptionally by EsaB, and is required for persistent abscess formation during animal infections [81]. Burt *et al.* 2008 found that expression of EsaC is tightly regulated in several *S. aureus* strains, with EsaC expression being up-regulated in the presence of serum [81]. The EsaC-like proteins of *S. mutans* range in size from 102 to 106 aa with a DUF4176 (pfam-domain of unknown function 4176) with very little homology to EsaC from *S. aureus* (Figure S8). Additionally there was an apparent homolog of the T7SS-associated protein, Lmo0069, of *Listeria monocytogenes* found in four of the strains with a T7SS (Table S1).

As noted above, a number of strains contained the *cnm* gene, which encodes a collagen binding protein [51]. Located directly upstream of the *cnm* gene are two ORFs that encode proteins with collagen binding-like domains, for convenience designated here as *cnaB*/*cbpA*/*cnm*. CbpA and Cnm both contain signal sequences for secretion (SignalP 4.0 [82]), whereas CnaB does not. The *cnm* gene sequence is incomplete for these strains (due to the repeat sequences at the C-terminus of the *cnm* gene) and only includes the coding sequence for the first 346 amino acids of the Cnm protein. The 120-kDa product of the *cnm* gene of *S. mutans* was first described by Sato *et al*. [51] and contains a collagen binding domain (pfam05737) preceding a putative B-region consisting of a tandem TTTTE(K/A)P followed by 19 TTTE(A/S/T)P repeats, an architecture common among MSCRAMMs (microbial surface components recognizing adhesive matrix molecules) [83]. The *cnm* gene was found in 12–20% of clinical isolates of *S. mutans* [84] and has been implicated in invasion of human coronary artery endothelial cells [9]. It was also shown that invasive strains containing the *cnm* gene were more virulent in the wax-worm *Galleria mellonella* [10]. The *cnaB* gene is predicted to encode a 61-kDa protein with conserved domains for collagen binding (pfam05737) and a Cna protein B-type domain (pfam05738) similar to Cnm, found in collagen binding proteins of *Staphylococcus aureus* (Figure S9) [85]. *cbpA* is predicted to encode a 53-kDa protein and also contains a conserved Cna protein B-type domain (pfam05738) (Figure S9) and shares 82% sequence identity with the collagen-binding A precursor protein-like protein from *Streptococcus ratti* FA-1 (EJN94659). To our knowledge this is the first time the *cnaB* and *cbpA* genes have been described in *S. mutans.*

In a recently sequenced *cnm*+ serotype *k* strain LJ23, a gene predicted to encode a protein (containing a signal sequence, collagen binding domain, Cna protein B-type domain and the LPTXG-cell wall anchoring motif) with 75% sequence similarity to CnaB (Figure S10) was described [73]. On further examination of the sequence from *S. mutans* LJ23, there are two possible open reading frames (ORF1 and ORF2) not annotated in NCBI, with homology to the N-terminus and C-terminus, respectively, of CbpA located upstream of the coding sequence for *cnm* (Figures S11 and S12). In all the strains sequenced in this study [34], the protein sequence (Figure S13) and gene arrangement of the *cnaB*, *cbpA* and *cnm* genes are almost identical.

**Figure 6:**
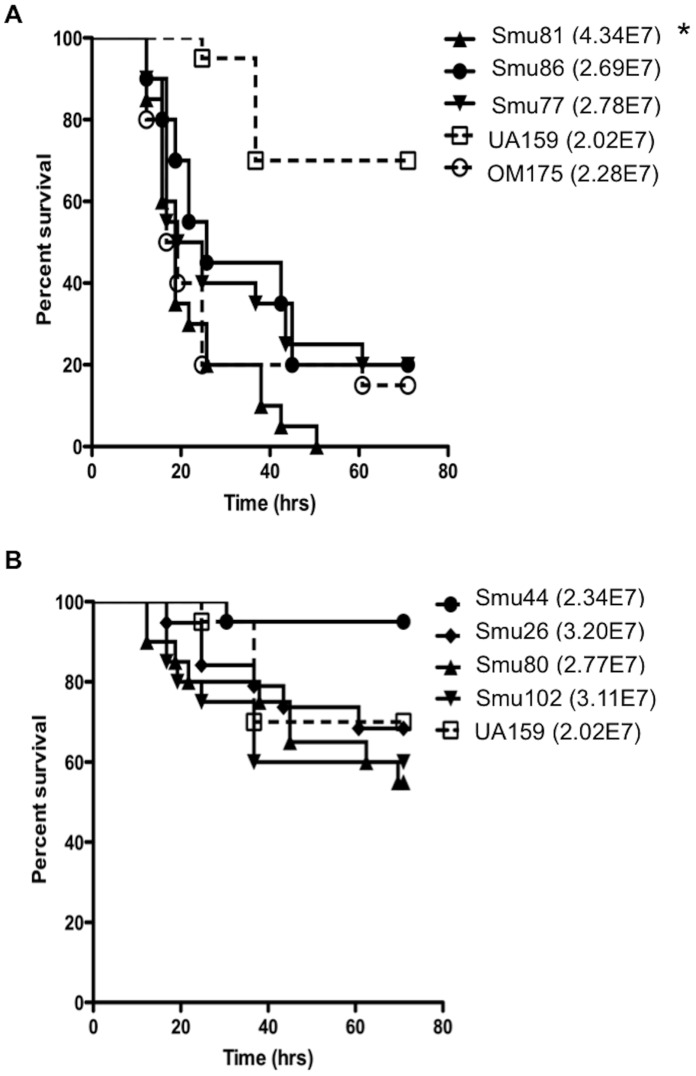
Kaplan-Meier killing curves of *G. mellonella* larvae (wax worms) inoculated with *S. mutans* strains. Graphs show percent survival of *G. mellonella* after injection of 5 µl inoculums. CFU/100 µl of each inoculum is provided in legend. A) Smu81, Smu86, Smu77 compared to UA159 (non-virulent) and OMZ175. B) Smu44, Smu26, Smu80 and Smu102 compared to UA159. * Indicates significant difference (*P≥*0.05) compared to OMZ175.

In order to determine what role the presence of these genes might play in the virulence of *S. mutans*, strains that contained the *cnaB*/*cbpB*/*cnm* collagen binding protein genes (Smu77/V1996, Smu81, Smu86 and Smu109/OMZ175) or the genes for the T7SS (Smu26, Smu44, Smu80 and Smu102) were assayed for virulence in the *G. mellonella* model (Figure 6). For comparison purposes, we also included the type strain UA159 (lacking both *cnaB-cbpA-cnm* and T7SS) and a *cnm* mutant of OMZ175 (OMZΔ*cnm*). As previously seen [10], OMZ175 was significantly more virulent than UA159 and inactivation of *cnm* strongly attenuated the virulence of OMZ175 (data not shown). Along these lines, strains Smu77, Smu81 and Smu86 expressing *cnaB*-*cbpA*-*cnm* were significantly more virulent than UA159 (Figure 6A). Strains Smu77/V1996 and Smu86 (serotypes *c* and *e*, respectively) were as virulent as Smu109 (OMZ175), a well-characterized serotype *f* strain [9], [86]–[89], whereas Smu81 killed *G. mellonella* more rapidly than OMZ175. These results support previous findings that *cnm* is an important virulence factor in the *G. mellonella* invertebrate model and furthermore, reveals that serotype *c* strains carrying genes for collagen binding proteins are equally as virulent compared to serotype *f* strains [9], [10]. On the other hand, strains that contained the genes for the T7SS did not display significantly greater virulence compared with UA159 (Figure 6B).

## Discussion

*S. mutans* is a genetically-diverse species that co-exists in the oral cavity with a number of other streptococci and hundreds of additional species of bacteria from a wide range of taxa [90], [91]. Contributing to its diversity is its ability to take up DNA from the environment allowing this organism to acquire new genes through lateral gene transfer, which may have enhanced the ability of *S. mutans* to adapt to and survive the selective pressures of the oral cavity as human diets became richer in refined sugars and polysaccharides [34], [92]. In this study, we demonstrate that phenotypic properties that have been directly associated with the ability of *S. mutans* to establish, persist, and/or cause disease in humans vary considerably among 15 genetically- and geographically-heterogeneous clinical isolates for which high coverage genome sequence is now available. The particular phenotypes analyzed here are complex, and multiple gene products contribute to the manifestation of, for example, growth at low pH or oxidative stress resistance. Importantly, this study, coupled with the genome sequencing information, which is publically available via a newly developed genome browser (http://strep-genome.bscb.cornell.edu), opens the way for evaluating relationships between gene content and virulence potential, and lays the foundation for determining how the core and non-core genomes interact to allow adaptation to the constantly-changing environment of the human oral cavity.

The genome sequences of these *S. mutans* strains reveal that approximately 1,490 genes constitute the core genome, while the pan-genome contains roughly 3,300 genes [34], which is substantial in comparison to the 1,963 ORFs found in UA159 [25]. The knowledge that so many non-core genes can be found in *S. mutans* underscores the limitations of using only one genome sequence as a reference for functional genomic studies and highlights the adaptive potential of this species for survival in the oral cavity. Population genetic analysis based on the core genes indicates that a large population expansion of *S. mutans* took place between 3,000 and 10,000 years ago, coinciding with the adoption of agriculture by humans and the associated expansion of the quantity and type of carbohydrates in the diet [34]. While agriculture and industrialization increased survival and influenced evolution of the human species, it also provided new selective pressures for the organisms in the human oral microbiome, resulting in the evolution of bacteria able to withstand the stresses induced by excess carbohydrates (e.g. acid production), a critical characteristic of *S. mutans*, while dramatically enhancing the prevalence of human dental caries [93]–[95].

The F_1_F_O_-H+-translocating ATPase is considered to be a major determinant of acid resistance in *S. mutans* [13]. The activity and pH optima of these ATPase enzymes are strongly correlated with acid tolerance in oral bacteria. For example, strongly aciduric organisms, such as lactobacilli, display much greater activity and lower pH optima for the ATPase than does the acid-sensitive species *Streptococcus sanguinis* [96]. Of interest, there was substantial variation in the ability of different *S. mutans* strains to contend with acid stress. In particular, several strains displayed faster doubling times during growth at pH 5.5 and showed increased survival at a killing pH of 2.8, compared to UA159. However, the enhanced acid resistance of these strains was not correlated with ATPase activity, which was similar in all strains and conditions tested, although all strains expressed higher ATPase activity after acid-adaptation. We did not directly examine the pH optima of the enzymes, but the F_1_F_o_ -ATPase subunits in all strains were very highly conserved (99.6% identical) with very few polymorphisms within individual subunits. Therefore, differences in membrane proton permeability or other physiological traits may account for the differences in acid tolerance between strains.

Interestingly, oxidative stress resistance showed the greatest spectrum of behaviors among the strains, with one strain (Smu81) displaying an unusually high degree of sensitivity to oxidative stress induced by the superoxide generator paraquat. The reason for paraquat sensitivity in Smu81 remains a mystery, as there are no obvious loci missing (e.g. superoxide dismutase) relevant to those pathways that have already been shown to contribute to oxidative stress resistance in other strains of *S. mutans.* While the presence of genes can clearly be determined with certainty, this is not necessarily so regarding the absence of genes in draft genome sequences. Thus, Smu81 may be lacking gene product(s) required for paraquat tolerance, or aberrantly expresses or contains key mutations in core genes that compromise its tolerance of paraquat. Transcriptomic studies are planned to address the former hypothesis.

A particularly interesting observation was the growth-lysis-regrowth phenotype displayed by several strains in the presence of air, which could indicate that a subset of isolates have adapted this strategy to cope with the stress induced by exposure to oxygen. *S. mutans* has several known mechanisms that mediate cell lysis, including bacteriocins (regulated by ComCDE), the autolysin AltA, and the apparent holin:antiholin complexes LrgAB and CidAB, which are regulated by oxygen and are growth phase dependent [39], [97], [98], that could account for the growth-lysis-regrowth phenotype. Furthermore, altruistic behavior of bacteria has been well documented, wherein programmed cell death (PCD) is used by organisms to ensure the survival of a population by sacrificing a sub-population during stress or when resources become limiting [55], [99]. It is therefore conceivable that these systems are regulated differently in some *S. mutans* strains depending on the genome content, and that the differences seen between strains grown in air are due to differential gene regulation in response to oxygen. Further analysis of the gene content, or of the transcriptomes of these strains in the presence of oxygen, might lead to the discovery of novel mechanisms for oxygen tolerance and alternative gene regulation pathways in *S. mutans* and other oral streptococci that regulate PCD. Notably, the growth-lysis-regrowth phenotype is probably not induced by superoxide radical alone, as the behavior is not observed in the presence of paraquat. Perhaps endogenous production of hydrogen peroxide [100] or hyper-expression of bacteriocins in response to oxygen [97] acts as a signal to trigger sub-population lysis.

The development of genetic competence in *S. mutans* is a complex process involving multiple inputs [101]–[104]. The accumulation of a processed form of the *comC* gene product (CSP) results in signaling through a phosphorylation cascade via a two-component system (ComD and ComE), which induces expression of bacteriocins and stimulates cells to take up exogenous DNA through mechanisms that are not yet completely understood [15], [59]. More recently, it was discovered that another peptide known as XIP, for *sigX*-inducing peptide, encoded by the *comS* gene also plays an important role in *comX* activation by modulating the DNA binding activity of the transcription factor ComR [105], [106]. Interestingly, *comR* and *comS* are found in all 57 strains sequenced, but significant numbers of the sequenced strains either lacked the *comCDE* genes or contained various mutations that could lead to failure to produce functional ComCDE proteins. There were a number of polymorphisms within the *comR* gene resulting in variation in the protein sequence (Figure S14), however there was no correlation of these polymorphisms with the competence phenotypes observed. On the other hand, the protein sequence for ComS was 100% identical in all 57 *S. mutans* strains sequenced. Polymorphisms within the *comCDE* locus of *S. mutans* isolates have been noted previously [45], resulting in variability in genetic competence, as well as pleiotropic effects associated with deletion of *comDE* in different *S. mutans* strains [101]. Interestingly, about one-fifth of the sequenced isolates encoded a ComC peptide with a C-terminal truncation of 3 amino acids (producing an 18-mer CSP peptide), while one-third of sequenced strains lacked *comC* all together. Although the presence of the 18-mer allele of CSP has been documented previously [36], [107], it was shown only recently that CSP is processed extracellularly at the C-terminus by a protease (SepM) to yield an 18-mer CSP peptide, and that C-terminal processing can be required for CSP signaling [108]. Currently the implications of the distribution of 21-mer and 18-mer CSP species across strains of *S. mutans* are unclear. However, these results reveal that both versions of CSP are common in the population of *S. mutans,* and that competence and competence-related phenotypes are largely independent of the allele of *comC*, which is consistent with studies detailed elsewhere [108]. Our results extend these findings and further demonstrate that tremendous variation exists among strains in the pathways involved in quorum sensing and genetic competence, and also that natural genetic competence can not be readily induced in a laboratory setting in many strains *of S. mutans.*

A survey of genes that encode non-lantibiotic bacteriocin and immunity proteins revealed a number of polymorphisms and disparities in the distribution of these genes in the strains described here. Of particular note are differences in the pathway thought to be responsible for CSP-induced lysis, with a significant number of strains missing the bacteriocin CipB/Smu.1914 (Smu21, Smu44, Smu.56, Smu.57, and Smu98) or the immunity protein CipI/Smu.925 (Smu86, Smu98 and Smu109) [60]. Additionally, in two strains (Smu77 and Smu81) there appears to be a recombination between Smu.1914/CipB and Smu.1906 resulting in a hybrid protein that contains the signal sequence of CipB and the bacteriocin sequence from Smu.1906. Furthermore, a number of strains contained mutations within the *cipI* gene sequence that resulted in either a truncation at the N-terminus of 18 amino acids (Smu20, Smu77, Smu81, Smu93 and Smu104) and/or a truncation of 4 amino acids (Smu20, Smu56, Smu77 and Smu104) or 7 amino acids at the C- terminus (Smu21). Recent work by Dufour [61] showed that the unprocessed CipB bacteriocin contributes to the CSP-dependent increase in transformation efficiency seen in UA159, in which a *ΔcipB* mutant showed no increase in transformants upon addition of CSP. Transcriptome analysis via microarray of a *ΔcipB* mutant revealed that CipB acts at the transcriptional level through regulation of ComE, ComR and ComX [61]. This study also found that deletion of *cipI* encoding the immunity protein to CipB results in increased expression of competence genes and increased transformation efficiency in the absence of exogenously-added CSP [61]. On the other hand, *nlmT/*Smu.286 and *nlmE*/Smu.287, required for transport of non-lantibiotic bacteriocins [109] are among the core genes of *S. mutans*. Considering the variation seen among isolates with respect to the presence or absence of competence genes, bacteriocins and immunity proteins, as well as the ability to become naturally competent, further study of multiple *S. mutans* strains is warranted in order to determine the roles that these gene products might play in competence and biofilm ecology in the context of different genetic backgrounds. Also, variations within the competence pathway tend to sort with genes related to bacteriocin production, which likely have evolved differently from other more highly conserved competence related genes and may therefore be of interest to the study of interspecies interactions, e.g. antagonism of commensal streptococci. Further still, the presence or absence of these gene products may reflect different adaptive strategies of the various isolates of *S. mutans*, and may be related to cariogenic potential as the differences could impact biofilm persistence and interactions with other members of the oral microbiome.

There was additional variability in the capacity of strains to form biofilms and on further examination this variation could be partially attributed to differences in Gtf isozyme production. Two strains, Smu20 and Smu44, which formed biofilms relatively poorly in sucrose medium, were found to have a recombination between *gtfB* and *gtfC*, as reported for some other strains of *S. mutans* [64]–[66]. Of note, there were also several strains lacking *gbpA*, the absence of which appeared to correlate with the presence of the *cnm* gene. Notably, Nakano *et al.* reported that 79% of strains containing the *cnm* gene lacked the *gbpA* gene [84]. Recently a new collagen binding protein, termed Cbm, was described in *S. mutans* clinical isolates [110], however this collagen binding protein is different from those described here. Also, the *cbm* gene was found in only 2% of strains [110] and does not appear to be present in any of the 57 isolates sequenced by our group. The serotype *f* strain, OMZ175 (referred to as Smu109 in this study), which has been well characterized for its invasiveness of endothelial cells and virulence in the *G. mellonella* model [9], [10], along with three additional strains, were found to contain two ORFs upstream of *cnm* that encode putative proteins with possible collagen binding functions (*cnaB*/*cbpA*/*cnm*). While the incidence of collagen binding proteins within the species *S. mutans* is relatively low, there seems to be an increased frequency in non-serotype *c* strains. Here we report the incidence of two serotype *c* (Smu77/V1996 and Smu81) and one serotype *e* strain (Smu86) that contain the *cnaB/cbpA/cnm* genes and show for the first time that they are as virulent in the *G. mellonella* model as a serotype *f* strain. Considering the invasive nature of strains that carry the *cnm* gene, collagen-binding proteins could have implications for systemic diseases caused by *S. mutans* [17] and further characterization of *cnaB* and *cbpA* should be pursued. It is also interesting to speculate that the increased frequency of *cnm* in serotype *f* and *k* strains along with decreased glucan-binding capacity due to a lack of *gbpA*, could be an indication of adaptation by *S. mutans* for survival outside the oral cavity, and could therefore represent a lineage of this pathogen that is better adapted to contributing to systemic diseases.

We also report for the first time the presence of a Type VII secretion system (T7SS) in a number of *S. mutans* clinical isolates. While the role that this pathway may play in the physiology or virulence of *S. mutans* is unknown at this time, its presence in 8 of 57 strains is an exciting discovery. Strains with a T7SS did not display increased virulence in *G. mellonella*, which could be due to a lack of expression under assay conditions or perhaps the system contributes to the physiology or virulence in ways that are not relevant in the insect model. Preliminary studies looking at invasion of HCAEC did not indicate increased ability to invade (unpublished data) compared to UA159 and deletion of the *esxA* gene did not seem to affect growth or stress tolerance in at least two strains tested so far (unpublished data). Similar results were seen in *Listeria monocytogenes*, where deletion of the ESAT-6/EsxA homolog had no effect on virulence and or growth [111]. However, in *L. monocytogenes*, only the *esxA* effector gene was inactivated, and therefore the possibility that other effectors secreted by the T7SS could compensate for its loss remains. Further dissection of the *S. mutans* T7SS may lead to the discovery of other effector proteins that are secreted through this pathway, which could have implications on survival of the organism in complex biofilms or in the circulatory system of the host.

The phenotypic characterization of *S. mutans* strains presented here, in conjunction with the WGS sequences of the 57 isolates [34], will provide a powerful set of resources to the *S. mutans* community, allowing for the discovery of new genes that contribute to the virulence of this caries pathogen. In addition, much insight into the evolution and phenotypic diversity of the *S. mutans* species as a whole in the context of genome-scale information has been gained. This study will allow researchers to compare the phenotypes of strains with the presence or absence of certain genes, and to take a more focused approach to functional genomics studies. Furthermore, this study will also provide a foundation for future analysis of core and non-core genome interactions. For example studies are currently underway in our laboratory to dissect the role in stress-tolerance of the “unique core” genes, which are a group of genes that is present in all *S. mutans* isolates but not in closely-related mutans streptococci [34]. Additionally, the genome sequence data combined with RNAseq will allow us to evaluate how different strains utilize their transcriptomes to cope with environmental stress and quorum sensing, leading to a better overall understanding of gene regulation in the streptococci. While the aim of this study was not to correlate the presence of *S. mutans* strains and or gene content with caries or virulence, our results can be used to form new hypotheses concerning gene content of the *S. mutans* species and may lead to a better understanding of how the species *S. mutans* is involved in the process of caries development, infective endocarditis and cardiovascular disease.

## Supporting Information

Figure S1 **Gene content differences between strains based on orthologs recovered across genomes via an all-versus-all BLASTP search combined with clustering using OrthoMCL2** [**46**] **.**
(TIFF)

Figure S2 **Growth-lysis-growth phenotype of select strains in the presence of oxygen stress (no mineral oil overlay).** Duplicate early exponential phase cultures were diluted 1∶100 into BHI and inoculated in duplicate wells of a Bioscreen C microtiter plate. Growth was monitored at 37°C every 30 min in a Bioscreen C labsystem for 48 hr.
(TIFF)

Figure S3 **Sequence alignment of** ***comE*** **gene from UA159 and Smu81.** The *comE* gene from Smu81 contains a frameshift at 409 nucleotides stemming from an insertion of two nucleotides, resulting in a mutated ComE protein.
(TIFF)

Figure S4 **Sequence alignment of** ***comC*** **gene from UA159 and Smu81.** The *comC* gene from Smu81 contains an extra adenosine four nucleotides into the DNA sequence, resulting in a frameshift and no ComC protein.
(TIFF)

Figure S5 **Sequence alignment of** ***comX*** **from Smu104 and UA159.** The *comX* gene from Smu104 contains a C to T mutation resulting in a stop codon 26 nucleotides into the *comX* gene.
(TIFF)

Figure S6 **Sequence alignment of** ***comC*** **gene from UA159 compared to Smu69 and Smu93.** The *comC* gene from Smu69 and Smu93 contain an insertion of 18 nucleotides resulting in a stop codon and a truncated 18-mer ComC peptide.
(TIFF)

Figure S7 **Sequence of inserted 4605 bp DNA within the** ***gtfD*** **gene of Smu77.** Blast results revealed that the DNA sequence is from the *Enterococcus faecalis* DS16 transposon Tn916 corresponding to ORF11-14, containing the gene sequence for TetM (Orf11), TetM leader peptide (Orf12), conjugative transposon protein TcpC (Orf13), and a protein containing a lysozyme domain that cleaves beta, 1–4, linked polysaccharides (Orf14).
(PDF)

Figure S8 **The four EsaC homologs from strain Smu44 compared to EsaC from** ***S. aureus*** **subspecies** ***aureus*** **JH9.** A) Protein sequence and size of EsaC homologs and contig location in Smu44. B) ClustalW2 sequence alignment of EsaC homologs.
(TIFF)

Figure S9 **Conserved domains of CnaB, CbpA, and Cnm as determined using NCBI’s CDD** [**85**] **.**
(TIFF)

Figure S10 **ClustalW2 sequence alignment of CnaB protein from Smu81 and CnaB homolog from** ***S. mutans*** **Serotype k strain LJ23.**
(TIFF)

Figure S11 **ClustalW2 sequence alignment of CbpA from Smu81 and ORF1 from** ***S. mutans*** **strain LJ23.**
(TIFF)

Figure S12 **ClustalW2 sequence alignment of CbpA from Smu81 and ORF2 from** ***S. mutans*** **strain LJ23.**
(TIFF)

Figure S13 **ClustalW sequence alignment of CnaB, CbpA and Cnm protein sequence from Smu109, Smu86, Smu81 and Smu77.** Signal sequence cleavage site for CbpA and Cnm as predicted by SignalP (version 4.0) is indicated *. LPXTG sequence for cell-wall anchoring is underlined for CnaB and CbpA. The Cnm gene sequence is incomplete.
(PDF)

Figure S14 **ClustalW sequence alignment of ComR protein sequence from all 15 strains.** Smu44, Smu56, Smu57, Smu69, Smu81, Smu98 and Smu104 are not competent for natural transformation.
(PDF)

Table S1 **Distribution of Type VII secretion genes among clinical isolates of** ***S. mutans.***
(PDF)
