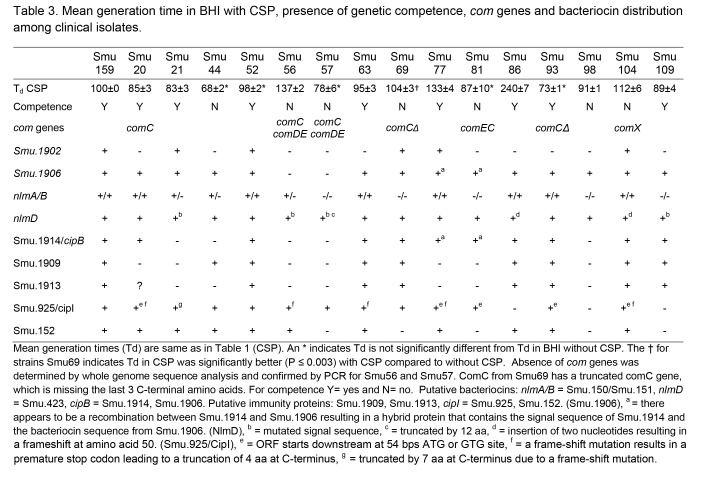# Correction: Phenotypic Heterogeneity of Genomically-Diverse Isolates of *Streptococcus mutans*

**DOI:** 10.1371/annotation/ffff8cd5-b8fa-4d3c-a993-e5169198f1e6

**Published:** 2013-11-08

**Authors:** Sara R. Palmer, James H. Miller, Jacqueline Abranches, Lin Zeng, Tristan Lefebure, Vincent P. Richards, José A. Lemos, Michael J. Stanhope, Robert A. Burne

In Table 3, multiple - (negative signs) were replaced by blank spaces. Please see the corrected Table 3 here: 

**Figure:**