# Supplementary figures and images for: Phenotypic Heterogeneity of Genomically-Diverse Isolates of Streptococcus mutans
Source: PLoS One. 2013 Apr 16;8(4):e61358. doi: 10.1371/journal.pone.0061358 (PMC3628994; doi:10.1371/journal.pone.0061358)

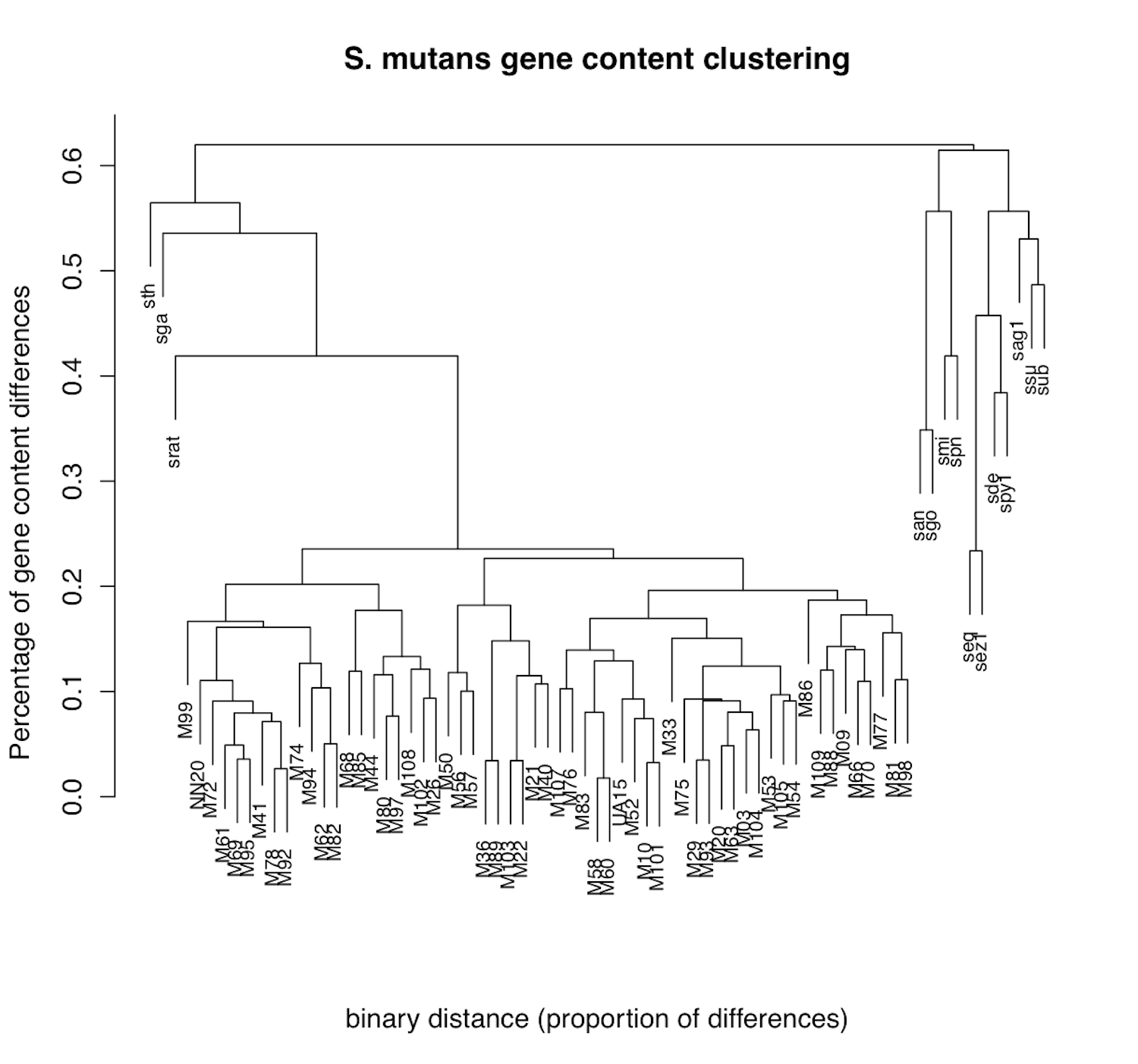

Supplement: Figure S1 — Gene content differences between strains based on orthologs recovered across genomes via an all-versus-all BLASTP search combined with clustering using OrthoMCL2 [46] . (TIFF) [file pone.0061358.s001.tiff]

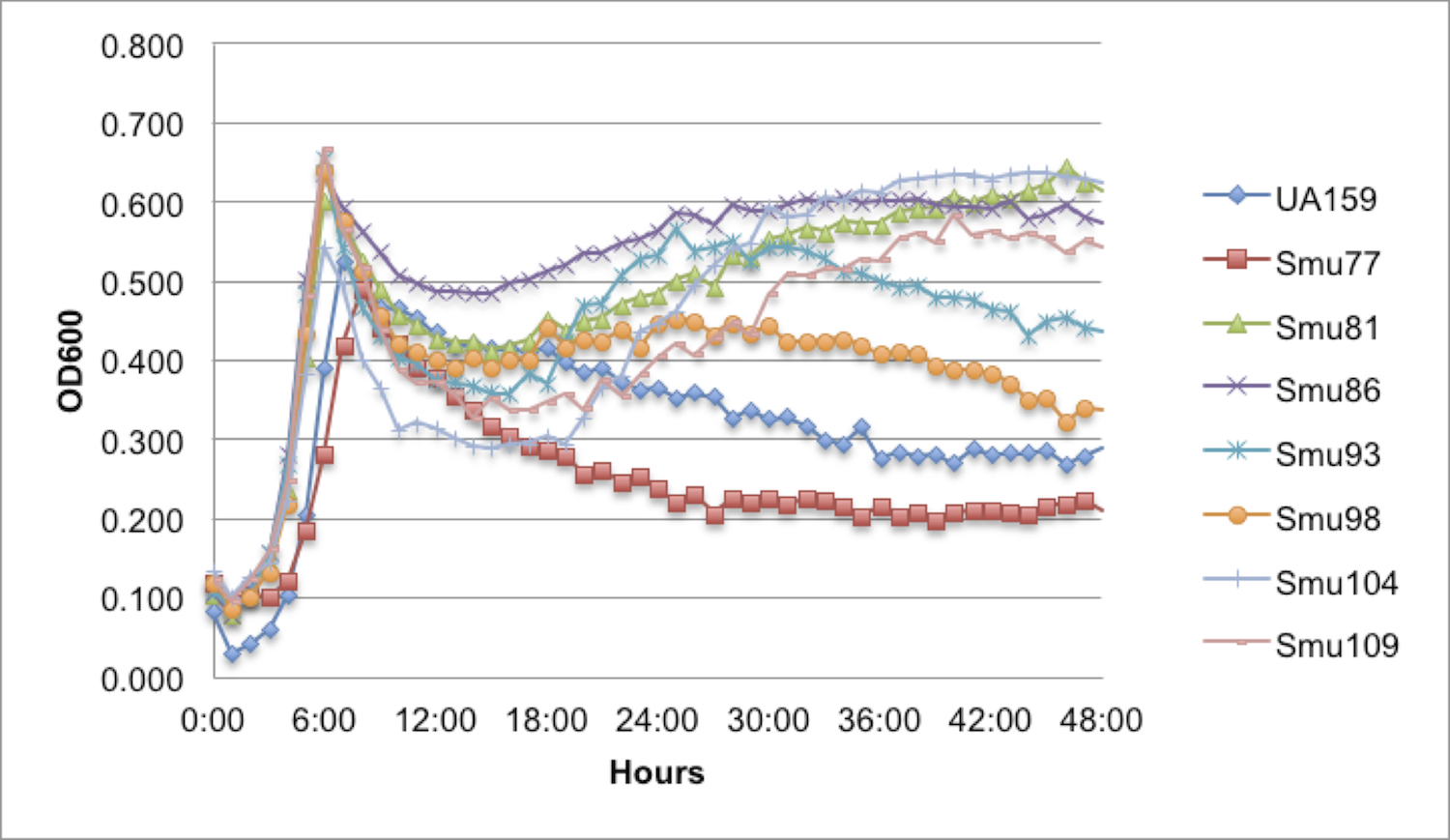

Supplement: Figure S2 — Growth-lysis-growth phenotype of select strains in the presence of oxygen stress (no mineral oil overlay). Duplicate early exponential phase cultures were diluted 1∶100 into BHI and inoculated in duplicate wells of a Bioscreen C microtiter plate. Growth was monitored at 37°C every 30 min in a Bioscreen C labsystem for 48 hr. (TIFF) [file pone.0061358.s002.tiff]

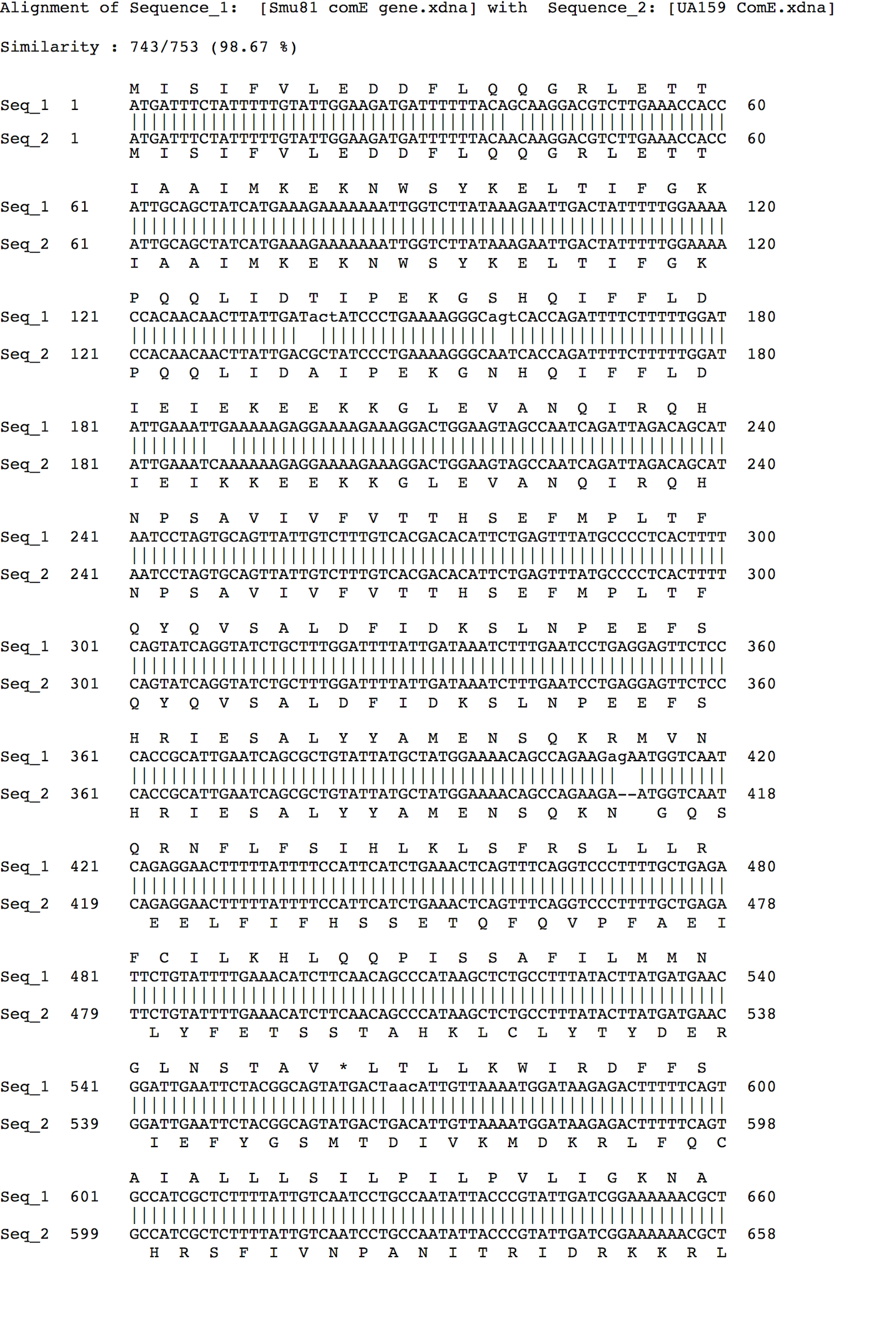

Supplement: Figure S3 — Sequence alignment of comE gene from UA159 and Smu81. The comE gene from Smu81 contains a frameshift at 409 nucleotides stemming from an insertion of two nucleotides, resulting in a mutated ComE protein. (TIFF) [file pone.0061358.s003.tiff]

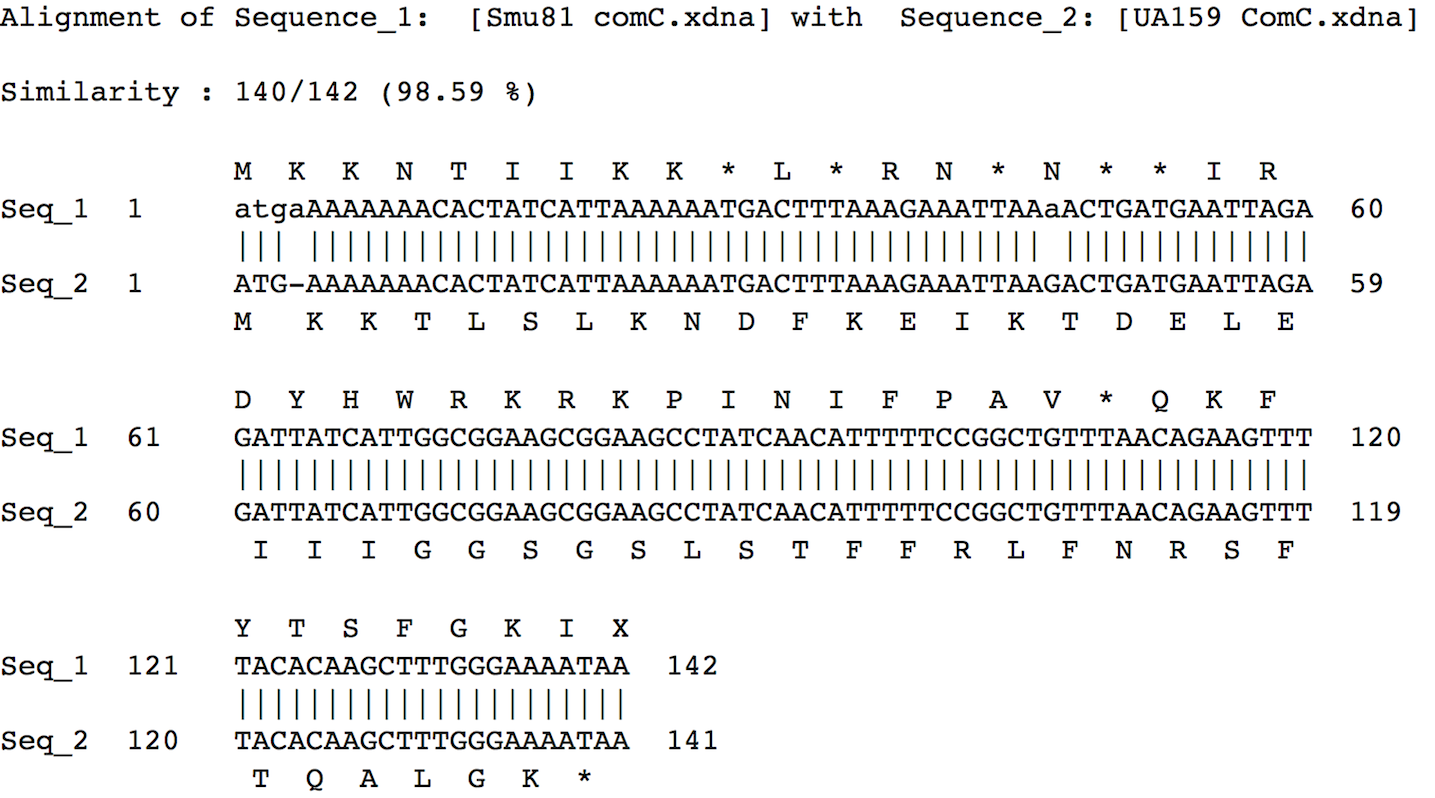

Supplement: Figure S4 — Sequence alignment of comC gene from UA159 and Smu81. The comC gene from Smu81 contains an extra adenosine four nucleotides into the DNA sequence, resulting in a frameshift and no ComC protein. (TIFF) [file pone.0061358.s004.tiff]

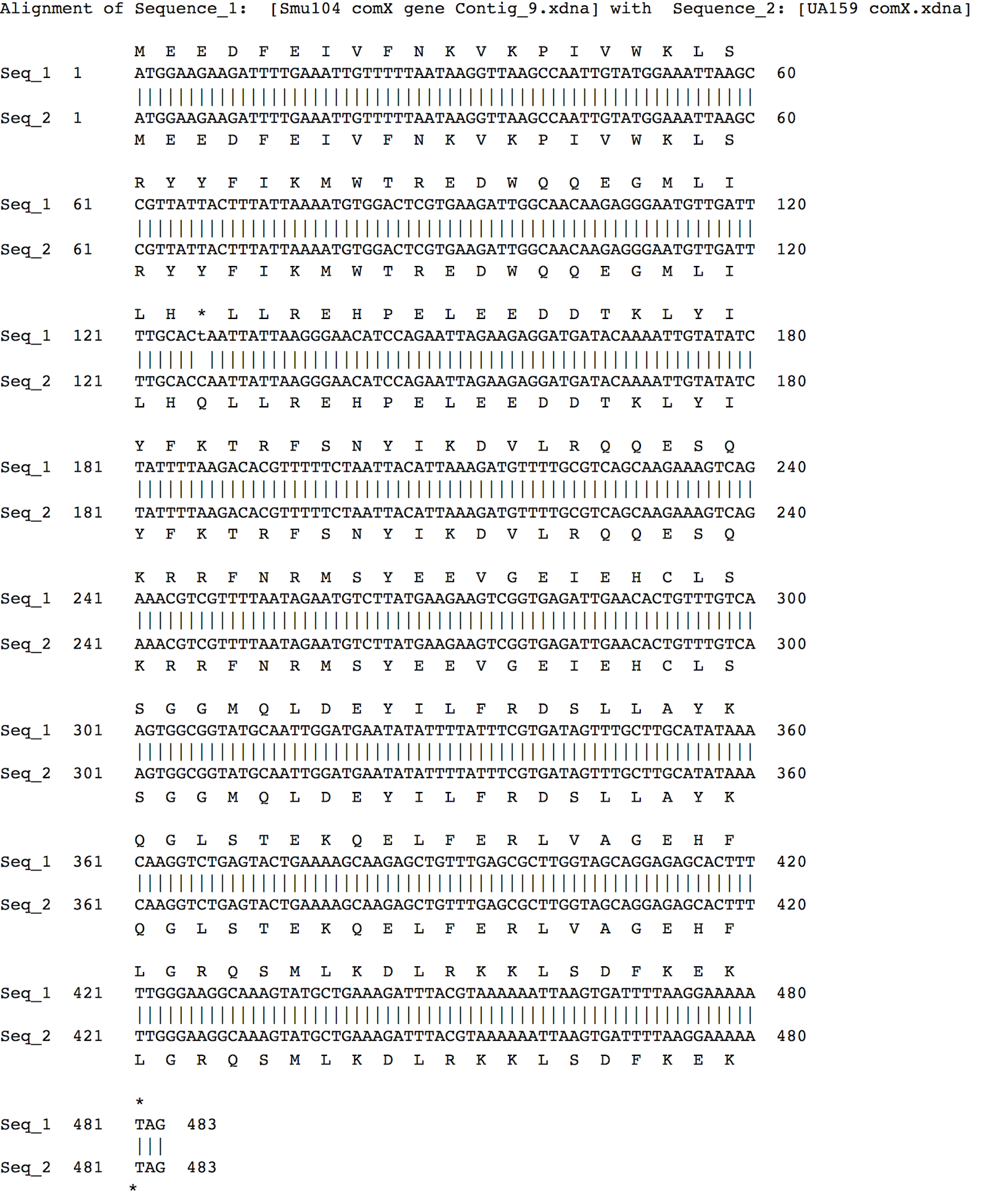

Supplement: Figure S5 — Sequence alignment of comX from Smu104 and UA159. The comX gene from Smu104 contains a C to T mutation resulting in a stop codon 26 nucleotides into the comX gene. (TIFF) [file pone.0061358.s005.tiff]

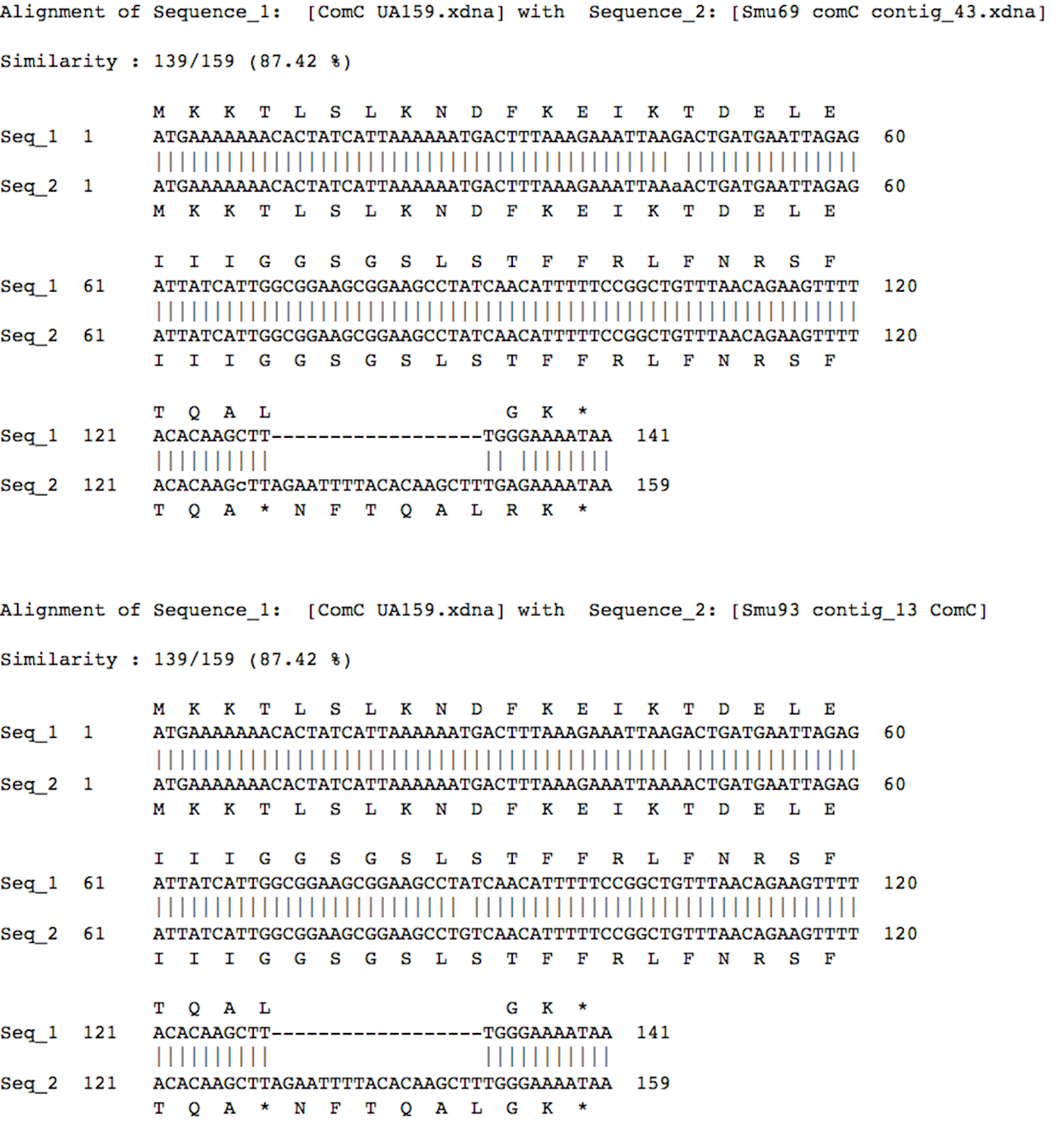

Supplement: Figure S6 — Sequence alignment of comC gene from UA159 compared to Smu69 and Smu93. The comC gene from Smu69 and Smu93 contain an insertion of 18 nucleotides resulting in a stop codon and a truncated 18-mer ComC peptide. (TIFF) [file pone.0061358.s006.tiff]

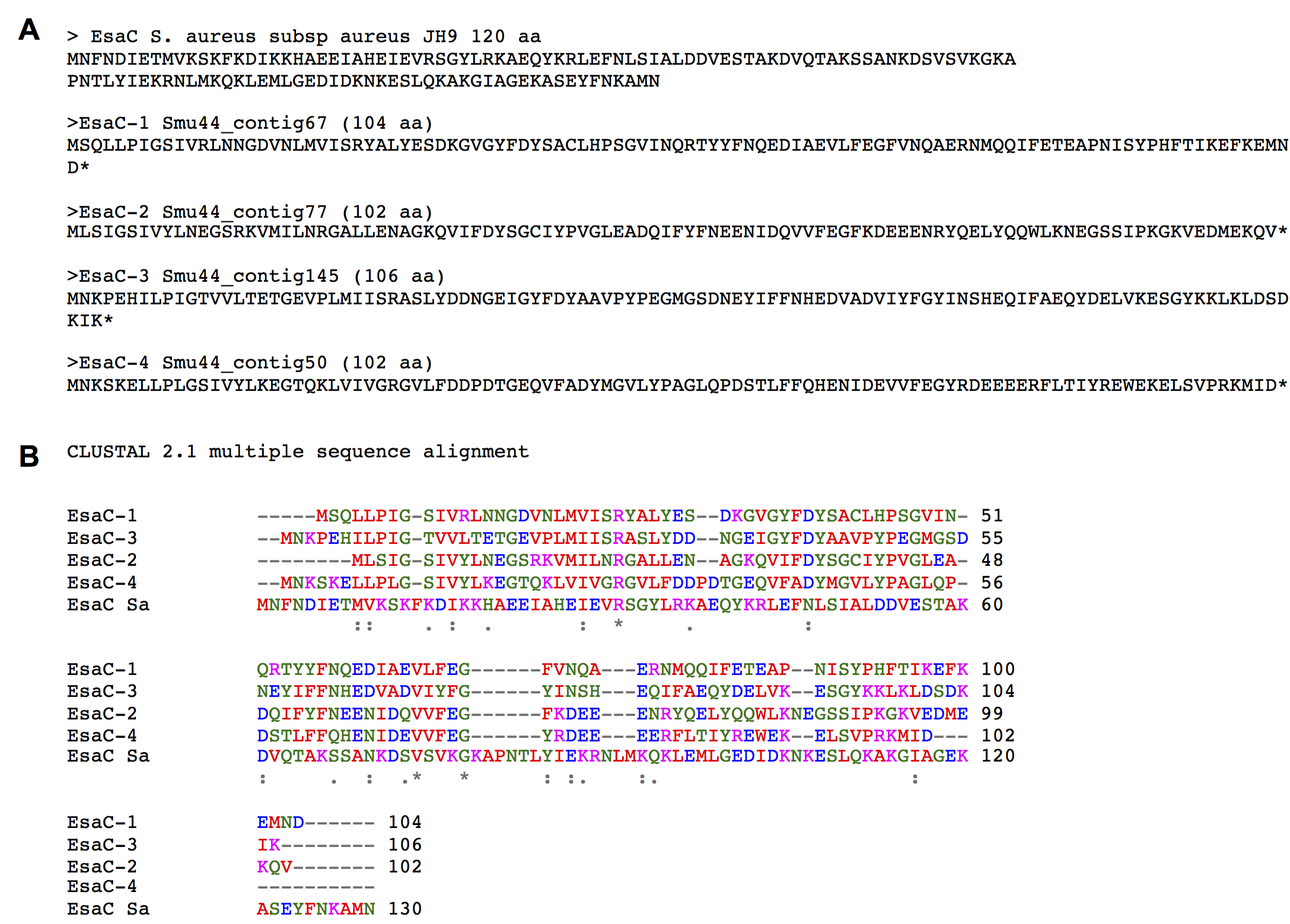

Supplement: Figure S8 — The four EsaC homologs from strain Smu44 compared to EsaC from S. aureus subspecies aureus JH9. A) Protein sequence and size of EsaC homologs and contig location in Smu44. B) ClustalW2 sequence alignment of EsaC homologs. (TIFF) [file pone.0061358.s008.tiff]

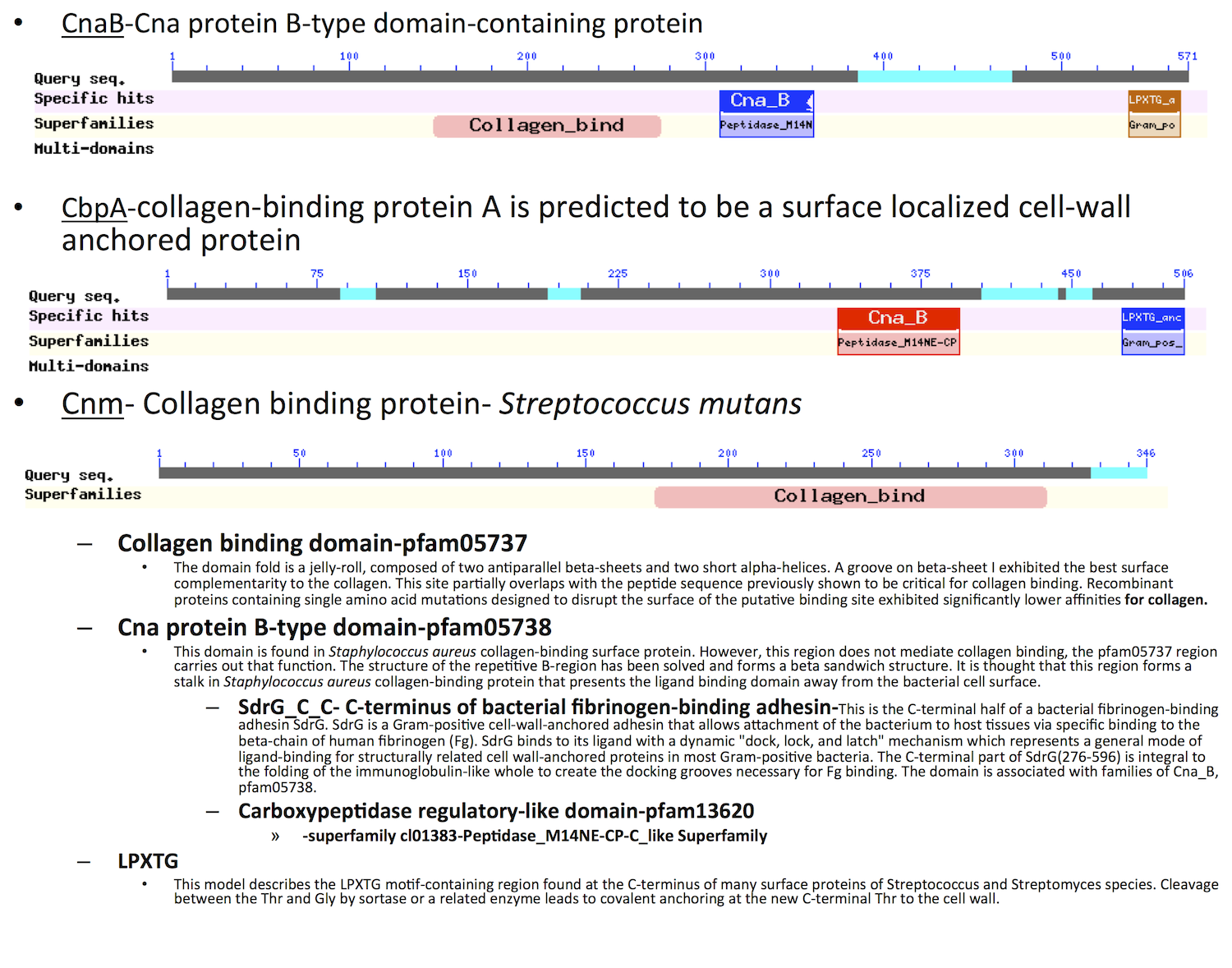

Supplement: Figure S9 — Conserved domains of CnaB, CbpA, and Cnm as determined using NCBI’s CDD [85] . (TIFF) [file pone.0061358.s009.tiff]

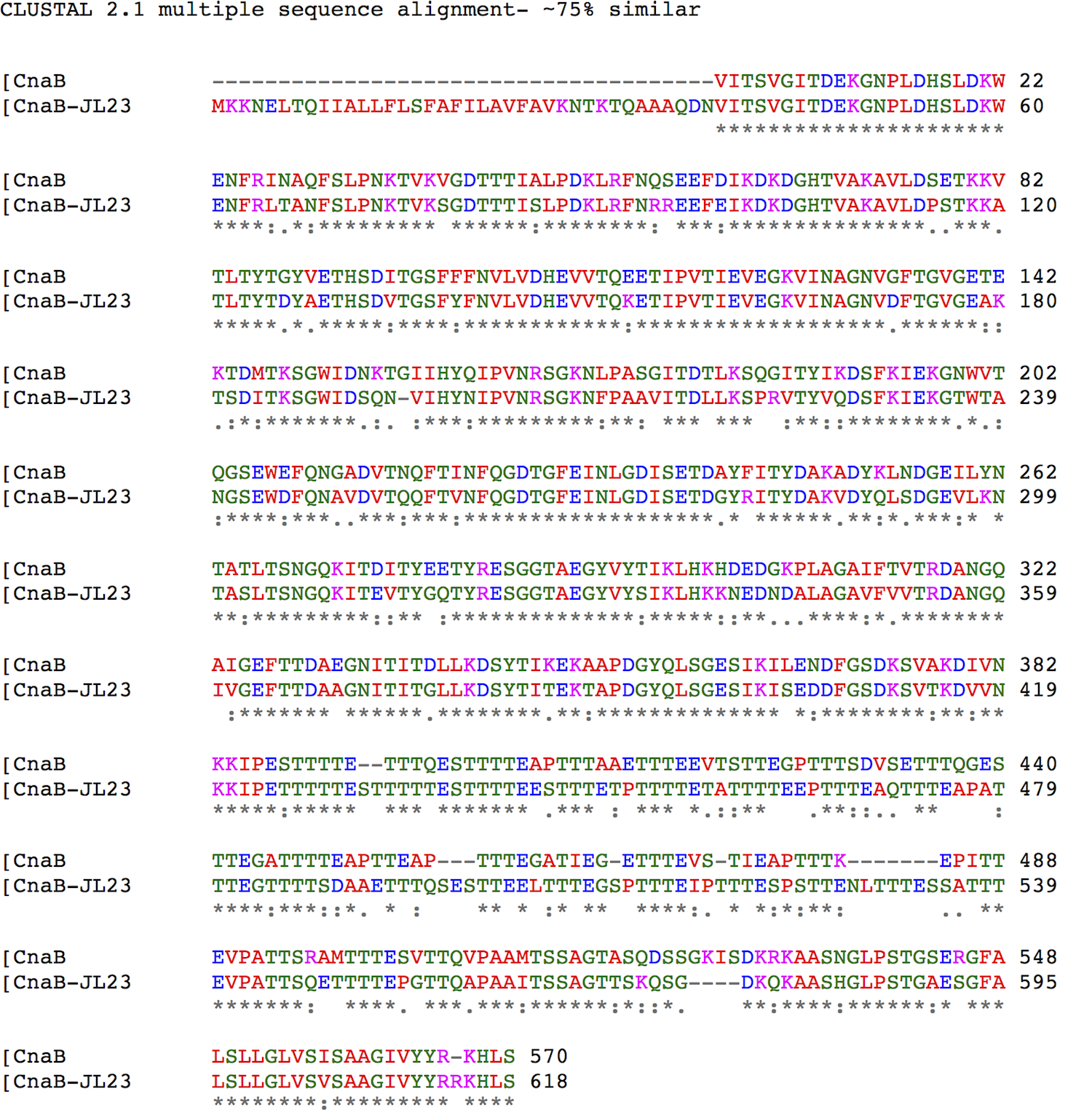

Supplement: Figure S10 — ClustalW2 sequence alignment of CnaB protein from Smu81 and CnaB homolog from S. mutans Serotype k strain LJ23. (TIFF) [file pone.0061358.s010.tiff]

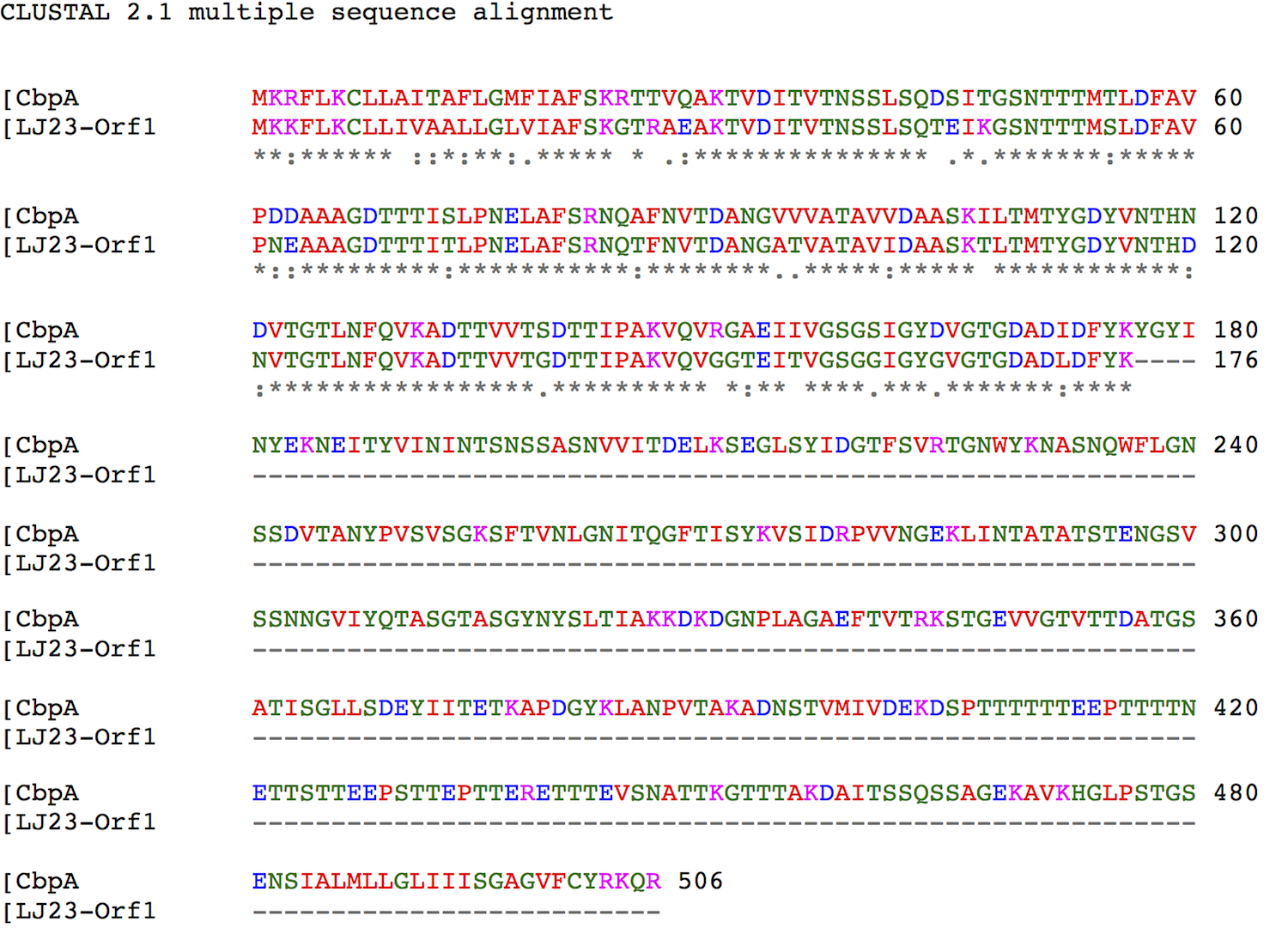

Supplement: Figure S11 — ClustalW2 sequence alignment of CbpA from Smu81 and ORF1 from S. mutans strain LJ23. (TIFF) [file pone.0061358.s011.tiff]

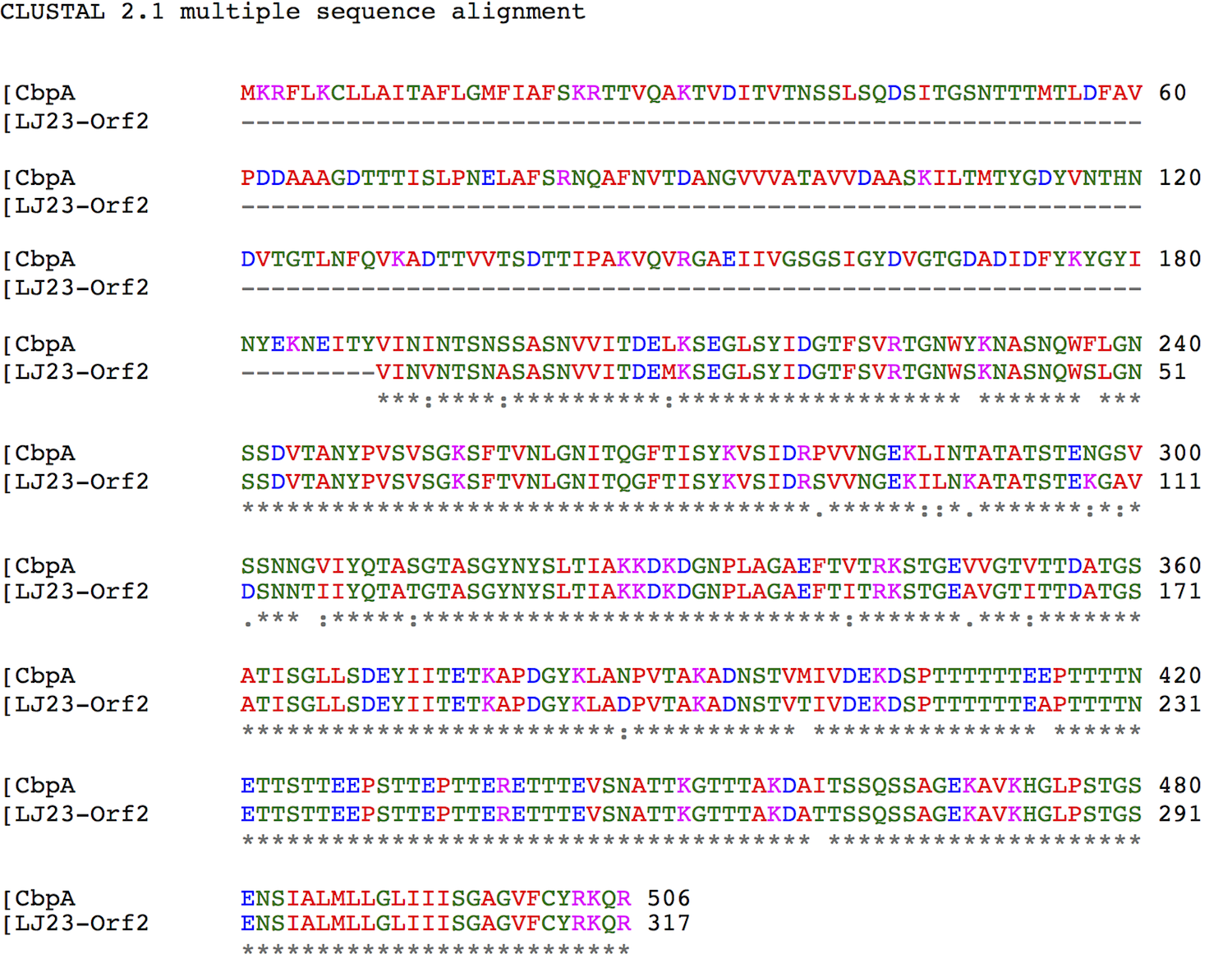

Supplement: Figure S12 — ClustalW2 sequence alignment of CbpA from Smu81 and ORF2 from S. mutans strain LJ23. (TIFF) [file pone.0061358.s012.tiff]
