# Supplementary material for: Phenotypic Heterogeneity of Genomically-Diverse Isolates of Streptococcus mutans
Source: PLoS One. 2013 Apr 16;8(4):e61358. doi: 10.1371/journal.pone.0061358 (PMC3628994; doi:10.1371/journal.pone.0061358)
Supplement: Figure S7 — Sequence of inserted 4605 bp DNA within the gtfD gene of Smu77. Blast results revealed that the DNA sequence is from the Enterococcus faecalis DS16 transposon Tn916 corresponding to ORF11-14, containing the gene sequence for TetM (Orf11), TetM leader peptide (Orf12), conjugative transposon protein TcpC (Orf13), and a protein containing a lysozyme domain that cleaves beta, 1–4, linked polysaccharides (Orf14). (PDF) [file pone.0061358.s007.pdf]

>gb|U09422.1|EFU09422 Enterococcus faecalis DS16 transposon Tn916, (tet(M)), (Xis-Tn), (Int-Tn) genes, ORFs 1-24, complete cds, complete sequence
Length=18032

 Score = 7825 bits (4237), Expect = 0.0
 Identities = 4237/4237 (100%), Gaps = 0/4237 (0%)

```
       caa tcatttttta actcgccatt atttatcggt ttcttttcaa  8034
tcaacatgtc tgcaatttct acggctacat ttaaagagtt tatatcatta  7984
tttaagcggt aatctacaag taaaggatat attaaacttc cttttccaaa  7934
aaaagctgac aatatatctt cagatggtat tgtataaatt gaatttttta  7884
tacattcaat tacatagtca tatttatgat tctttgtaat gtatttaaga  7834
gccacataaa atatgaatat accaggcaat ccatcgtaca tattattatt  7784
taggattccc acattccaat cttgatccaa ctttatgtca atccaattta  7734
cagtattggt ttttacataa aattttcttg tctgcatttc ggacaataga  7684
gggggaattt ttttaattca gtatcttccc ttatctttaa tcgtgtttta  7634
tttccacata caggacacaa tatccacttg tagtttataa taactatctc  7584
ctcctttaca ctttaattca aatctttatt aaaaaatatt tcatcttatt  7534
taacaagaaa ccatatttat ataacaacat aaaatacact aagttatttt  7484
attgaacata tatcgtactt tatctatccg actatttgga cgacggggct  7434
ggcaaacagg ttcaccggta gtaacatggt accctttaa ctctgttaaa  7384
caaacactac gtccatttgt aaagaaagtt aaatcactac gatattcttg  7334
aatacaccga gcagggattt ctccactaag aatgacctca ttattttca  7284
attgagtgtc tacgatgttc gcacaatatt taggagcatc gttgtatgct  7234
cgtgaaagat attcctgtgg cgcataaatt ttaaaactaa gatatggctc  7184
taacaattct gttccagctt tttttaagac ttgttccaat acaataggag  7134
caagcatccg aaaatctgct ggggtactaa cagggctata gtataagcca  7084
tacttaaaac agattttaca gtccgtcaca ttccaaccat acaatccttg  7034
ttcacaacca tagcgtatcc cttccataac tgcattttga aatgattgat  6984
ttaagtatcc aagagaaacc gagctctcat actgcattcc acttcccaac  6934
ggaagcggtg atacagataa accaatggaa gcccagaaag gatttggcgg  6884
cacttcgatg tgaatggtat attctgcatt ttttaacggt ctctccatat  6834
aaatgactgt aggctctttt agttctatct ccacatgata cttttcttgc  6784
aacagtgcac taatcacttc catttgtact ttccctaaga aagaaagtat  6734
aatttcatgt gtcgtagaat ccacgtaata tcgtagaagc ggatcactat  6684
ctgagatttc caaaagggca tcaagcaaca tttctctctg ttcaggttta  6634
ctcggttcaa cagttgtttg tagtagaggg tgcggatttt caatcttttt  6584
tctctgtggc aatagttttg tatctccaag aacactattt aacttcaaaa  6534
actcattttg caaaataaca atttctccag aataagctct atcaatctta  6484
cataattcac catttattga agtatacatt tctgtaactt ttattttttc  6434
tttttctgat actctaaccg aatctcgtaa atgtagtact ccactataaa  6384
ggcgtatata tgcaagacgt tgtctttttt ttgtatattc aattttgaaa  6334
acatttccgc aaagttcaga cggacctcga tgtgttgatg aataaaattt  6284
attagtaata acttctataa ggttatcaat ccctatatta ctttttgcac  6234
ttccatgata aagagggaac agagaacaat tctgaaatct tatgctttcc  6184
tcttgttcga gttccaatgc ttctaatgat ttaccggaca tatatttctc  6134
taaaaggtca tcgtttccct ctattaccgt atcccattgt tcagattcgg  6084
taaagttcgt cacacacaca ttaggataca gttctacctt ctgtttgatt  6034
acaatttcgg cagaaagttt ctctttaata tcctgataaa ccgttgataa  5984
atcaattcca ttttggtcaa tcttattgat aaaaaagatt gtgggaatcc  5934
ccatttttcct aagtgcatga aataatatac gagtttgtgc ttgtacgcca  5884
tcttttgcag aaatcagtag aattgcccca tctaaaactg ataatgaacg  5834
atatacttct gctaagaaat ccatatgtcc tggcgtgtct atgatgttca  5784
ccttcgtatt ttcccactga aaagaggtta ttcctgtctg aattgtaatt  5734
cctctctgac gttctaaaag cgtattatcc gtcctcgttg tacctttgtc  5684
```

```
cacgcttcct aattctgtaa tcgctccact gttatataat aagctttctg  5634
ttaaggtagt ttttcctgca tcaacatgag ctaaaactcc aatattaata  5584
attttcatgt gattttcctc cattcaaaag cccaaaaggg cataaaaatc  5534
ccagtgataa atactcttat cactgggatt tttatgcata accataggca  5484
tacaaagcat acagatattc tccggatact ttagaatcac atgataaagg  5434
tattcttaaa ctgggtacaa aaaactaagc cctcctaaaa aaggacatcc  5384
aattatttgt tcccgctatc aaattgacag tttatttaat aataccttgc  5334
cgcatattta ttaactcctt ttaaatagat acttaaataa tagcacgtaa  5284
gagcatattt gtaaaggaat ctccaatttt ttatcaaaga gagtacgtga  5234
ttacaaaata gctgtaataa tgtaccaata tttgttattc tataatcttc  5184
caattactcc cgttcttttc aagtaccaaa tcaaattgag atacctgcgt  5134
tgctttggtc tgctggtcga tatactccac tgtcagcgat accgtgactt  5084
gattatcctt acgattgtga ataggattta ccagttcttg aaagatgtac  5034
tcttttccga ttggttttaa tatcccgtca ttcacatagt aggaaagttc  4984
actggctgtc gctgtaggat agagcttgaa gaacgtcgtt aaaaactcat  4934
tgatttcatt ggttgtaatg gaatcaaccg tcccctcact ttcaatggct  4884
tttggtttat aacttgattt cttaggtatg ttggtaatgg tcggattctt  4834
aaccagtacc atatttccag aaccatctac atagacactc actatataag  4784
cagagtggac ggtctttgta ttttctccct ctgtaatgag ctggtctaca  4734
ctgtaggtta cattaaactc attgtcgcca gttggctcta ccgtccatat  4684
ctgaaatcct cttacagaag acgatacagg aatatctttg cgtactgtat  4634
caacattgag agcttgaagt tcatctgtca gatagccttt tagactttcc  4584
attcgattat caatggactt atcggattgc tcccatgaat agtagacttt  4534
cgcaaagttc tctacaaaat tttctacatg atgagtatca acgtattcct  4484
tttctatgat agttgtttcg tgaatagtat gagtatctat agctgtaaag  4434
tgcttgaata tcgcaaagct gaaactaagc cctaaaagta cccacaaggc  4384
aatcacaacc tttttatgag gattgacctt atagacacga ggtttctttt  4334
cctttggtat ctgtttttct ttattctgat tttttctaaa tttcatcatt  4284
aaatcttcct ttctcattgt ttgattcgtc ctgctcccac taaatgctgt  4234
tgccagtagg ggcttgttaa gtcggcataa ccgattgggt cgcctgcatg  4184
aaacatacgg ttattgccaa ggtatatccc aacatgagta atataagagc  4134
cagcgttata ggtagaatga aagaaaacca aatcgccagc ttgtgcttcc  4084
gatagtggga tatgctgggt cacatcatat tgctgttgtg cggttcgtgg  4034
taagttaatt ccagcttttc catacgtcca ttgtgtcagt ccgctacaat  3984
caaaagaagt agtcggggaa gctccaccgt aaacgtatcg ccagccctca  3934
tatttcagtg cttcgtccat gatggcttgt accgtatcat catcaaactc  3884
tgttgtgaca agatactgcg ttaccagttg cacataaaac atattgccat  3834
agttgtatcg ccagccccca ttgataggta tggctatggg attggggtaa  3784
gacacttttt cgccacctga atactctttt gagaaacttt gagccagttc  3734
aaaggtatat ttatttccac gattagccac ataccctaag aaaccaccac  3684
cataattgta ggactggata accgattcta aatctacact gagcctttcg  3634
ctactggcta ataattcact gaaatacttc acaccttgct taatggattc  3584
ttctgtactc aatgaattag gtggaagacc gagggattcc gaggactgca  3534
taacatcttc cgcagtaccg cccgattcca cctgtataat cgcaagaagt  3484
atgttgggga tc
```
