# Supplementary material for: Phenotypic Heterogeneity of Genomically-Diverse Isolates of Streptococcus mutans
Source: PLoS One. 2013 Apr 16;8(4):e61358. doi: 10.1371/journal.pone.0061358 (PMC3628994; doi:10.1371/journal.pone.0061358)
Supplement: Figure S13 — ClustalW sequence alignment of CnaB, CbpA and Cnm protein sequence from Smu109, Smu86, Smu81 and Smu77. Signal sequence cleavage site for CbpA and Cnm as predicted by SignalP (version 4.0) is indicated *. LPXTG sequence for cell-wall anchoring is underlined for CnaB and CbpA. The Cnm gene sequence is incomplete. (PDF) [file pone.0061358.s013.pdf]

CnaB

```
M109|SMU109_07971      VITSVGITDEKGNPLDHSLDKWENFRINAQFSLPNKTVKVGDTTTIALPDKLRFNQSEEF 60
M77|SMU77_06742        VITSVGITDEKGNPLDHSLDKWENFRINAQFSLPNKTVKVGDTTTIALPDKLRFNQSEEF 60
M81|SMU81_06633        VITSVGITDEKGNPLDHSLDKWENFRINAQFSLPNKTVKVGDTTTIALPDKLRFNQSEEF 60
M86|SMU86_07865        VITSVGITDEKGNPLDHSLDKWENFRINAQFSLPNKTVKVGDTTTIALPDKLRFNQSEEF 60
                       ************************************************************

M109|SMU109_07971      DIKDKDGHTVAKAVLDSETKKVTLTYTGYVETHSDITGSFFFNVLVDHEVVTQEETIPVT 120
M77|SMU77_06742        DIKDKDGHTVAKAVLDSETKKVTLTYTGYVETHSDITGSFFFNVLVDHEVVTQEETIPVT 120
M81|SMU81_06633        DIKDKDGHTVAKAVLDSETKKVTLTYTGYVETHSDITGSFFFNVLVDHEVVTQEETIPVT 120
M86|SMU86_07865        DIKDKDGHTVAKAVLDSETKKVTLTYTGYVETHSDITGSFFFNVLVDHEVVTQEETIPVT 120
                       ************************************************************

M109|SMU109_07971      IEVEGKVINAGNVGFTGVGETEKTDMTKSGWIDNKTGIIHYQIPVNRSGKNLPASGITDT 180
M77|SMU77_06742        IEVEGKVINAGNVGFTGVGETEKTDMTKSGWIDNKTGIIHYQIPVNRSGKNLPASGITDT 180
M81|SMU81_06633        IEVEGKVINAGNVGFTGVGETEKTDMTKSGWIDNKTGIIHYQIPVNRSGKNLPASGITDT 180
M86|SMU86_07865        IEVEGKVINAGNVGFTGVGETEKTDITKSGWIDNKTGIIHYQIPVNRSGKNLPASGITDT 180
                       *************************:**********************************

M109|SMU109_07971      LKSQGITYIKDSFKIEKGNWVTQGSEWEFQNGADVTNQFTINFQGDTGFEINLGDISETD 240
M77|SMU77_06742        LKSQGITYIKDSFKIEKGNWVTQGSEWEFQNGADVTNQFTINFQGDTGFEINLGDISETD 240
M81|SMU81_06633        LKSQGITYIKDSFKIEKGNWVTQGSEWEFQNGADVTNQFTINFQGDTGFEINLGDISETD 240
M86|SMU86_07865        LKSQGITYIKDSFKIEKGNWVTQGSEWEFQNGADVTNQFTINFQGDTGFEINLGDISETD 240
                       ************************************************************

M109|SMU109_07971      AYFITYDAKADYKLNDGEILYNTATLTSNGQKITDITYEETYRESGGTAEGYVYTIKLHK 300
M77|SMU77_06742        AYFITYDAKADYKLNDGEILYNTATLTSNGQKITDITYEETYRESGGTAEGYVYTIKLHK 300
M81|SMU81_06633        AYFITYDAKADYKLNDGEILYNTATLTSNGQKITDITYEETYRESGGTAEGYVYTIKLHK 300
M86|SMU86_07865        AYFITYDAKADYKLNDGEILYNTATLTSNGQKITDITYEETYRESGGTAEGYVYTIKLHK 300
                       ************************************************************

M109|SMU109_07971      HDEDGKPLAGAIFTVTRDANGQAIGEFTTDAEGNITITDLLKDSYTIKEKAAPDGYQLSG 360
M77|SMU77_06742        HDEDGKPLAGAIFTVTRDANGQAIGEFTTDAEGNITITDLLKDSYTIKEKAAPDGYQLSG 360
M81|SMU81_06633        HDEDGKPLAGAIFTVTRDANGQAIGEFTTDAEGNITITDLLKDSYTIKEKAAPDGYQLSG 360
M86|SMU86_07865        HDEDGKPLAGAIFTVTRDANGQAIGEFTTDAEGNITITDLLKDSYTIKEKAAPDGYQLSG 360
                       ************************************************************

M109|SMU109_07971      ESIKILENDFGSDKSVAKDIVNKKIPESTTTTETTTQESTTTTEAPTTTAAETTTEEVTS 420
M77|SMU77_06742        ESIKILENDFGSDKSVAKDIVNKKIPESTTTTETTTQESTTTTEAPTTTAAETTTEEVTS 420
M81|SMU81_06633        ESIKILENDFGSDKSVAKDIVNKKIPESTTTTETTTQESTTTTEAPTTTAAETTTEEVTS 420
M86|SMU86_07865        ESIKILENDFGSDKSVAKDIVNKKIPESTTTTETTTQESTTTTEAPTTTAAETTTEEVTS 420
                       ************************************************************

M109|SMU109_07971      TTEGPTTTSDVSETTTQGESTTEGATTTTEAPTTEAPTTTEGATIEGETTTEVSTIEAPT 480
M77|SMU77_06742        TTEGPTTTSDVSETTTQGESTTEGATTTTEAPTTEAPTTTEGATIEGETTTEVSTIEAPT 480
M81|SMU81_06633        TTEGPTTTSDVSETTTQGESTTEGATTTTEAPTTEAPTTTEGATIEGETTTEVSTIEAPT 480
M86|SMU86_07865        TTEGPTTTSDVSETTTQGESTTEGATTTTEAPTTEAPTTTEGATIEGETTTEVSTIEAPT 480
                       ************************************************************

M109|SMU109_07971      TTKEPITTEVPATTSRAMTTTESVTTQVPAAMTSSAGTASQDSSGKISDKRKAASNGLPS 540
M77|SMU77_06742        TTKEPITTEVPATTSRAMTTTESVTTQVPAAMTSSAGTASQDSSGKISDKRKAASNGLPS 540
M81|SMU81_06633        TTKEPITTEVPATTSRAMTTTESVTTQVPAAMTSSAGTASQDSSGKISDKRKAASNGLPS 540
M86|SMU86_07865        TTKEPITTEVPATTSRAMTTTESVTTQVPAAMTSSAGTASQDSSGKISDKRKAASNGLPS 540
                       ************************************************************

M109|SMU109_07971      TGSERGFALSLLGLVSISAAGIVYYRKHLS 570
M77|SMU77_06742        TGSERGFALSLLGLVSISAAGIVYYRKHLS 570
M81|SMU81_06633        TGSERGFALSLLGLVSISAAGIVYYRKHLS 570
M86|SMU86_07865        TGSERGFALSLLGLVSISAAGIVYYRKHLS 570
                       ******************************
```

CbpA

```
M81|SMU81_06628        MKRFLKCLLAITAFLGMFIAFSKRTTVQA*KTVDITVTNSSLSQDSITGSNTTTMTLDFAV 60
M86|SMU86_07860        MKRFLKCLLAITAFLGMFIAFSKRTTVQA*KTVDITVTNSSLSQDSITGSNTTTMTLDFAV 60
M109|SMU109_07966      MKRFLKCLLAITAFLGMFIAFSKRTTVQA*KTVDITVTNSSLSQDSITGSNTTTMTLDFAV 60
M77|SMU77_06747        MKRFLKCLLAITAFLGMFIAFSKRTTVQA*KTVDITVTNSSLSQDSITGSNTTTMTLDFAV 60
                       ***************************** *******************************


M81|SMU81_06628        PDDAAAGDTTTISLPNELAFSRNQAFNVTDANGVVVATAVVDAASKILTMTYGDYVNTHN 120
M86|SMU86_07860        PDDAAAGDTTTISLPNELAFSRNQAFNVTDANGVVVATAVVDAASKILTMTYGDYVNTHN 120
M109|SMU109_07966      PDDAAAGDTTTISLPNELAFSRNQAFNVTDANGVVVATAVVDAASKILTMTYGDYVNTHN 120
M77|SMU77_06747        PDDAAAGDTTTISLPNELAFSRNQAFNVTDANGVVVATAVVDAASKILTMTYGDYVNTHN 120
                       ************************************************************

M81|SMU81_06628        DVTGTLNFQVKADTTVVTSDTTIPAKVQVRGAEIIVGSGSIGYDVGTGDADIDFYKYGYI 180
M86|SMU86_07860        DVTGTLNFQVKADTTVVTSDTTIPAKVQVRGAEIIVGSGSIGYDVGTGDADIDFYKYGYI 180
M109|SMU109_07966      DVTGTLNFQVKADTTVVTSDTTIPAKVQVRGAEIIVGSGSIGYDVGTGDADIDFYKYGYI 180
M77|SMU77_06747        DVTGTLNFQVKADTTVVTSDTTIPAKVQVRGAEIIVGSGSIGYDVGTGDADIDFYKYGYI 180
                       ************************************************************

M81|SMU81_06628        NYEKNEITYVININTSNSSASNVVITDELKSEGLSYIDGTFSVRTGNWYKNASNQWFLGN 240
M86|SMU86_07860        NYEKNEITYVININTSNSSASNVVITDELKSEGLSYIDGTFSVRTGNWYKNASNQWFLGN 240
M109|SMU109_07966      NYEKNEITYVININTSNSSASNVVITDELKSEGLSYIDGTFSVRTGNWYKNASNQWFLGN 240
M77|SMU77_06747        NYEKNEITYVININTSNSSASNVVITDELKSEGLSYIDGTFSVRTGNWYKNASNQWFLGN 240
                       ************************************************************

M81|SMU81_06628        SSDVTANYPVSVSGKSFTVNLGNITQGFTISYKVSIDRPVVNGEKLINTATATSTENGSV 300
M86|SMU86_07860        SSDVTANYPVSVSGKSFTVNLGNITQGFTISYKVSIDRPVVNGEKLINTATATSTENGSV 300
M109|SMU109_07966      SSDVTANYPVSVSGKSFTVNLGNITQGFTISYKVSIDRPVVNGEKLINTATATSTENGSV 300
M77|SMU77_06747        SSDVTANYPVSVSGKSFTVNLGNITQGFTISYKVSIDRPVVNGEKLINTATATSTENGSV 300
                       ************************************************************

M81|SMU81_06628        SSNNGVIYQTASGTASGYNYSLTIAKKDKDGNPLAGAEFTVTRKSTGEVVGTVTTDATGS 360
M86|SMU86_07860        SSNNGVIYQTASGTASGYNYSLTIAKKDKDGNPLAGAEFTVTRKSTGEVVGTVTTDATGS 360
M109|SMU109_07966      SSNNGVIYQTASGTASGYNYSLTIAKKDKDGNPLAGAEFTVTRKSTGEVVGTVTTDATGS 360
M77|SMU77_06747        SSNNGVIYQTASGTASGYNYSLTIAKKDKDGNPLAGAEFTVTRKSTGEVVGTVTTDATGS 360
                       ************************************************************

M81|SMU81_06628        ATISGLLSDEYIITETKAPDGYKLANPVTAKADNSTVMIVDEKDSPTTTTTTEEPTTTTN 420
M86|SMU86_07860        ATISGLLSDEYIITETKAPDGYKLANPVTAKADNSTVMIVDEKDSPTTTTTTEEPTTTTN 420
M109|SMU109_07966      ATISGLLSDEYIITETKAPDGYKLANPVTAKADNSTVMIVDEKDSPTTTTTTEEPTTTTN 420
M77|SMU77_06747        ATISGLLSDEYIITETKAPDGYKLVNPVTAKADNSTVMIVDEKDSPTTTTTTEEPTTTTN 420
                       ************************.***********************************

M81|SMU81_06628        ETTSTTEEPSTTEPTTERETTTEVSNATTKGTTTAKDAITSSQSSAGEKAVKHGLPSTGS 480
M86|SMU86_07860        ETTSTTEEPSTTEPTTERETTTEVSNATTKGTTTAKDAITSSQSSAGEKAVKHGLPSTGS 480
M109|SMU109_07966      ETTSTTEEPSTTEPTTERETTTEVSNATTKGTTTAKDAITSSQSSAGEKAVKHGLPSTGS 480
M77|SMU77_06747        ETTSTTEEPSTTEPTTERETTTEVSNATTKGTTTAKDAITSSQSSAGEKAVKHGLPSTGS 480
                       ************************************************************

M81|SMU81_06628        ENSIALMLLGLIIISGAGVFCYRKQR 506
M86|SMU86_07860        ENSIALMLLGLIIISGAGVFCYRKQR 506
M109|SMU109_07966      ENSIALMLLGLIIISGAGVFCYRKQR 506
M77|SMU77_06747        ENSIALMLLGLIIISGAGVFCYRKQR 506
                       **************************
```

Cnm

```
M109|SMU109_07961      MKRKGLRRLLKFFGTVAIILPMFFIALTKAQA*SDVSNNVSSLTVSPTQINDGGKTTVRFE 60
M77|SMU77_06752        MKRKGLRRLLKFFGTVAIILPMFFIALTKAQA*SDVSNNVSSLTVSPTQINDGGKTTVRFE 60
M86|SMU86_10291        MKRKGLRRLLKFFGTVAIILPMFFIALTKAQA*SDVSNNVSSLTVSPTQINDGGKTTVRFE 60
M81|SMU81_06623        MKRKGLRRLLKFFGTVAIILPMFFIALTKAQA*SDVSNNVSSLTVSPTQINDGGKTTVRFE 60
                       ******************************** ****************************

M109|SMU109_07961      FDEHAQNIKAGDTITVNWQNSGTVRGTGYTKTIKLEVQGKYVGDLVVTQDKAVVTFNDSI 120
M77|SMU77_06752        FDEHAQNIKAGDTITVNWQNSGTVRGTGYTKTIKLEVQGKYVGDLVVTQDKAVVTFNDSI 120
M86|SMU86_10291        FDEHAQNIKAGDTITVNWQNSGTVRGTGYTKTIKLEVQGKYVGDLVVTQDKAVVTFNDSI 120
M81|SMU81_06623        FDEHAQNIKAGDTITVNWQNSGTVRGTGYTKTIKLEVQGKYVGDLVVTQDKAVVTFNDSI 120
                       ************************************************************

M109|SMU109_07961      TGLQNITGWGEFEIEGRNFTDTTTGNTGSFQVTSGGKTSEVTVVKSASGTTGVFYYKTGD 180
M77|SMU77_06752        TGLQNITGWGEFEIEGRNFTDTTTGNTGSFQVTSGGKTSEVTVVKSASGTTGVFYYKTGD 180
M86|SMU86_10291        TGLQNITGWGEFEIEGRNFTDTTTGNTGSFQVTSGGKTSEVTVVKSASGTTGVFYYKTGD 180
M81|SMU81_06623        TGLQNITGWGEFEIEGRNFTDTTTGNTGSFQVTSGGKTSEVTVVKSASGTTGVFYYKTGD 180
                       ************************************************************

M109|SMU109_07961      MQTDDTNHVRWFLNINNENAYVDSDIRIEDDIQSGQTLDIDSFDITVNGSESYRGQEGIN 240
M77|SMU77_06752        MQTDDTNHVRWFLNINNENAYVDSDIRIEDDIQSGQTLDIDSFDITVNGSESYRGQEGIN 240
M86|SMU86_10291        MQTDDTNHVRWFLNINNENAYVDSDIRIEDDIQSGQTLDIDSFDITVNGSESYRGQEGIN 240
M81|SMU81_06623        MQTDDTNHVRWFLNINNENAYVDSDIRIEDDIQSGQTLDIDSFDITVNGSESYRGQEGIN 240
                       ************************************************************

M109|SMU109_07961      QLAQRYGATISADSASGHISVYIPQGYASLNSFSIMYLTKVDNPDQKTFENNSKAWYKEN 300
M77|SMU77_06752        QLAQRYGATISADSASGHISVYIPQGYASLNSFSIMYLTKVDNPDQKTFENNSKAWYKEN 300
M86|SMU86_10291        QLAQRYGATISADSASGHISVYIPQGYASLNSFSIMYLTKVDNPDQKTFENNSKAWYKEN 300
M81|SMU81_06623        QLAQRYGATISADSASGHISVYIPQGYASLNSFSIMYLTKVDNPDQKTFENNSKAWYKEN 300
                       ************************************************************

M109|SMU109_07961      GKDAVDGKEFNHSVANVNAAGGVDGRTTTTTEKPTTTTEAPTTTEA- 346
M77|SMU77_06752        GKDAVDGKEFNHSVANVNAAGGVDGRTTTTTEKPTTTTEAPTTTEA- 346
M86|SMU86_10291        GKDAVDGKEFNHSVANVNAAGGVDGRTTTTTEKPTTTTEAPTTTEAP 347
M81|SMU81_06623        GKDAVDGKEFNHSVANVNAAGGVDGRTTTTTEKPTTTTEAPTTTEAP 347
                       **********************************************
```
