# Supplementary material for: Phenotypic Heterogeneity of Genomically-Diverse Isolates of Streptococcus mutans
Source: PLoS One. 2013 Apr 16;8(4):e61358. doi: 10.1371/journal.pone.0061358 (PMC3628994; doi:10.1371/journal.pone.0061358)
Supplement: Figure S14 — ClustalW sequence alignment of ComR protein sequence from all 15 strains. Smu44, Smu56, Smu57, Smu69, Smu81, Smu98 and Smu104 are not competent for natural transformation. (PDF) [file pone.0061358.s014.pdf]

ClustalW. Sequence alignment of ComR from S. mutans isolates.

```
UA159               MLKDFGKKIKSLRLEKGLTKEAVCLDESQLSTRQLTRIESGQSTPTLNKAVYIAGRLGVT 60
SMU20_07973         MLKDFGKKIKSLRLEKGLTKEAVCLDESQLSTRQLTRIESGQSTPTLNKAVYIAGRLGVT 60
SMU21_00120         MLKDFGKKIKSLRLEKGLTKEAVCRDESQLSIRQLTRIESGQSTPTLNKAVYIAGRLGVT 60
SMU44_05115         MLKDFGKKIKSLRLEKGLTKEAVCLDESQLSTRQLTRIESGQSTPTLNKAVYIAGRLGVT 60
SMU52_07301         MLKDFGKKIKSLRLEKGLTKEAVCLDESQLSTRQLTRIESGQSTPTLNKAVYIAGRLGVT 60
SMU56_00582         MLKDFGKKIKSLRLEKGLTKEAVCLDESQLSTRQLTRIESGQSVPTLNKAIYIAGRLGVT 60
SMU57_07778         MLKDFGKKIKSLRLEKGLTKEAVCLDESQLSTRQLTRIESGQSTPTLNKAVYIAGRLGVT 60
SMU63_06475         MLKDFGKKIKSLRLEKGLTKEAVCLDESQLSTRQLTRIESGQSTPTLNKAVYIAGRLGVT 60
SMU69_05470         MLKDFGKKIKSLRLEKGLTKEAVCLDESQLSTRQLTRIESGQSTPTLNKAVYIAGRLGVT 60
SMU77_05920         MLKDFGKKIKNLRLEKGLTKEAVCRDESQLSIRQLTRIESGQSTPTLNKAVYIAGRLGVT 60
SMU81_05044         MLKDFGKKIKSLRLEKGLTKEAVCLDESQLSTRQLTRIESGQSTPTLNKAVYIAGRLGVT 60
SMU86_02980         MLKDFGKKIKSLRLEKGLTKEAVCFDESQLSTRQLTRIESGQSMPTLNKAVYIAGRLGVT 60
SMU93_06493         MLKDFGKKIKSLRLEKGLTKEAVCLDESQLSTRQLTRIESGQSMPTLNKAIYIAGRLGVT 60
SMU98_07471         MLKDFGKKIKSLRLEKGLTKEAVCLDESQLSTRQLTRIESGQSTPTLNKAVYIAGRLGVT 60
SMU104_00375        MLKDFGKKIKSLRLEKGLTKEAVCRDESQLSIRQLTRIESGQSTPTLNKAVYIAGRLGVT 60
SMU109_08657        MLKDFGKKIKSLRLEKGLTKEAVCLDESQLSTRQLTRIESGQSMPTLNKAVYIAGRLGVT 60
                    **********.************* ****** *********** ******:*********

UA159               LGYLTDGENVELPSRYKELKYLLLRTPTYGDQQRLAEKETYFDEIFSQFYDDLPEEEQLI 120
SMU20_07973         LGYLTDGENVELPSRYKELKYLLLRTPTYGDQQRLAEKETYFDEIFSQFYDDLPEEEQLI 120
SMU21_00120         LGYLTDGENVELPSRYKELKYLLLRTPTYGDQQRLAEKETYFDEIFSQFYDDLPEEEQLI 120
SMU44_05115         LGYLTDGENVELPSRYKELKYLLLRTPTYGDQQRLAEKETYFDEIFSQFYDDLPEEEQLI 120
SMU52_07301         LGYLTDGENVELPSRYKELKYLLLRTPTYGDQQRLAEKETYFDEIFSQFYDDLPEEEQLI 120
SMU56_00582         LGYLTDGENVELPSRYKELKYLLLRTPTYGDQQRLAEKETYFDEIFSQFYDDLPEEEQLI 120
SMU57_07778         LGYLTDGENVELPSRYKELKYLLLRTPTYGDQQRLAEKETYFDEIFSQFYDDLPEEEQLI 120
SMU63_06475         LGYLTDGENVELPSRYKELKYLLLRTPTYGDQQRLAEKETYFDEIFSQFYDDLPEEEQLI 120
SMU69_05470         LGYLTDGENVELPSRYKELKYLLLRTPTYGDQQRLAEKETYFDEIFSQFYDDLPEEEQLI 120
SMU77_05920         LGYLTDGENVELPSRYKELKYLLLRTPTYGDQQRLAEKETYFDEIFSQFYDDLPEEEQLI 120
SMU81_05044         LGYLTDGENVELPSRYKELKYLLLRTPTYGDQQRLAEKETYFDEIFSQFYDDLPEEEQLI 120
SMU86_02980         LGYLTDGENVELPSRYKELKYLLLRTPTYGDQQRLAEKETYFDEIFSQFYDDLPEEEQLI 120
SMU93_06493         LGYLTDGENVELPSRYKELKYLLLRTPTYGDQQRLAEKETYFDEIFSQFYDDLPEEEQLI 120
SMU98_07471         LGYLTDGENVELPSRYKELKYLLLRTPTYGDQQRLAEKETYFDEIFSQFYDDLPEEEQLI 120
SMU104_00375        LGYLTDGENVELPSRYKELKYLLLRTPTYGDQQRLAEKETYFDEIFSQFYDDLPEEEQLI 120
SMU109_08657        LGYLTDGENVELPSRYKELKYLLLRTPTYGDQQRLAEKETYFDEIFSQFYDDLPEEEQLI 120
                    ************************************************************

UA159               IDGLQSKLDIHFSDNIDFGVGILNDYFDQILRKTNYQVNDLILIDLYFSCLTVSGLDSAI 180
SMU20_07973         IDGLQSKLDIHFSDNIDFGVGILNDYFDQILRKTNYQVNDLILIDLYFSCLTVSGLDSAI 180
SMU21_00120         IDGLQSKLDIHFSDNIDFGVGILNDYFDQILRKTNYQVNDLILIDLYFSCLTVSGLDSAI 180
SMU44_05115         IDGLQSKLDIHFSDNIDFGVGILNDYFDQILRKTNYQVNDLILIDLYFSCLTVSGLDSAI 180
SMU52_07301         IDGLQSKLDIHFSDNIDFGVGILNDYFDQILRKTNYQVNDLILIDLYFSCLTVSGLDSAI 180
SMU56_00582         IDGLQSKLDIHFSDNIDFGVGILNDYFDQILRKTNYQVNDLILIDLYFSCLTVSGLDSAI 180
SMU57_07778         IDGLQSKLDIHFSDNIDFGVGILNDYFDQILRKTNYQVNDLILIDLYFSCLTVSGLDSAI 180
SMU63_06475         IDGLQSKLDIHFSDNIDFGVGILNDYFDQILRKTNYQVNDLILIDLYFSCLTVSGLDSAI 180
SMU69_05470         IDGLQSKLDIHFSDNIDFGVGILNDYFDQILRKTNYQVNDLILIDLYFSCLTVSGLDSAI 180
SMU77_05920         IDGLQSKLDIHFSDNIDFGVGILNDYFDQILRKTNYQVNDLILIDLYFSCLTVSGLDSAI 180
SMU81_05044         IDGLQSKLDIHFSDNIDFGVGILNDYFDQILRKTNYQVNDLILIDLYFSCLTVSGLDSAI 180
SMU86_02980         IDGLQSKLDIHFSDNIDFGVGILNDYFDQILRKTNYQVNDLILIDLYFSCLTVSGLDSAI 180
SMU93_06493         IDGLQSKLDIHFSDNIDFGVGILNDYFDQILRKTNYQVNDLILIDLYFSCLTVSGLDSAI 180
SMU98_07471         IDGLQSKLDIHFSDNIDFGVGILNDYFDQILRKTNYQVNDLILIDLYFSCLTVSGLDSAI 180
SMU104_00375        IDGLQSKLDIHFSDNIDFGVGILNDYFDQILRKTNYQVNDLILIDLYFSCLTVSGLDSAI 180
SMU109_08657        IDGLQSKLDIHFSDNIDFGVGILNDYFDQILRKTNYQVNDLILIDLYFSCLTVSGLDSAI 180
                    ************************************************************

UA159               FDSRKYNQLLETLLKQVDCLPLEDLFVLNNVLLNNFGLLLELKKYDFVKQLIAVSNKIMD 240
SMU20_07973         FDSRKYNQLLETLLKQVDCLPLEDLFVLNNVLLNNFGLLLELKKYDFVKQLIAVSNKIMD 240
SMU21_00120         FDSRKYNQLLETLLKQVDCLPLEDLFVLNNVLLNNFGLLLELKKYDFVKQLIAVSNEIMA 240
SMU44_05115         FDSRKYNQLLETLLKQVDCLPLEDLFVLNNVLLNNFGLLLELKKYDFVKQLIAVSNKIMD 240
SMU52_07301         FDSRKYNQLLETLLKQVDCLPLEDLFVLNNVLLNNFGLLLELKKYDFVKQLIAVSNKIMD 240
SMU56_00582         FDLRKYNQLLETLLKQVDCLPLEDLFVLNNVLLNNFGLLLELKKYDFVKQLIAVSNKIMD 240
SMU57_07778         FDSRKYNQLLETLLKQVDCLPLEDLFVLNNVLLNNFGLLLELKKYDFVKQLIAVSNKIMT 240
SMU63_06475         FDSRKYNQLLETLLKQVDCLPLEDLFVLNNVLLNNFGLLLELKKYDFVKQLIAVSNKIMD 240
SMU69_05470         FDSRKYNQLLETLLKQVDCLPLEDLFVLNNVLLNNFGLLLELKKYDFVKQLIAVSNKIMA 240
SMU77_05920         FDSRKYNQLLETLLKQVDCLPLEDLFVLNNVLLNNFGLLLELKKYDFVKQLIAVSNKIMA 240
SMU81_05044         FDSRKYNQLLETLLKQVDCLPLEDLFVLNNVLLNNFGLLLELKKYDFVKQLIAVSNKIMA 240
SMU86_02980         FDSRKYNQLLETLLKQVDCLPLEDLFVLNNVLLNNFGLLLELKKYDFVKQLIAVSNKIMA 240
SMU93_06493         FDSRKYNQLLETLLKQVDCLPLEDLFVLNNVLLNNFGLLLELKKYDFVKQLIAVSNEIMA 240
SMU98_07471         FDSRKYNQLLETLLKQVDCLPLEDLFVLNNVLLNNFGLLLELKKYDFVKQLIAVSNKIMA 240
SMU104_00375        FDSRKYNQLLETLLKQVDCLPLEDLFVLNNVLLNNFGLLLELKKYDFVKQLIAVSNKIMA 240
SMU109_08657        FDSRKYNQLLETLLKQADCLPLEDLFVLNNVLLNNFGLLLELKKYDFVKQLIAVSNKIMA 240
                    ** *************.***************************************:** 
```

```
UA159            RTHDFQKKPIVNLLTWKHHLFVEKDYAEAKKSYDAAILFAQLTENINLRENLEKEWQKDS 300
SMU20_07973      RTHDFQKKPIVNLLTWKHHLFVEKDYAKAKKSYDAAILFAQLTENINLRENLEKEWQKDS 300
SMU21_00120      RTHDFQKKPIVNLLTWKHHLFVEKDYAKAKKSYDAAILFAQLTENINLRENLEKEWQKDS 300
SMU44_05115      RTHDFQKKPIVNLLTWKHHLFVEKDYAEAKKSYDAAILFAQLTENINLRENLEKEWQKDS 300
SMU52_07301      RTHDFQKKPIVNLLTWKHHLFVEKDYAEAKKSYDAAILFAQLTENINLRENLEKEWQKDS 300
SMU56_00582      RTHDFQKKPIVNLLTWKHHLFVEKDYAKAKKSYDAAILFAQLTENINLRENLEKEWQKDS 300
SMU57_07778      RTHDFQKKPIVNLLTWKHHLFVEKNYAEAKKSYDAAILFAQLTENINLRENLEKEWQKDS 300
SMU63_06475      RTHDFQKKPIVNLLTWKHHLFVEKDYAEAKKSYDAAILFAQLTENINLRENLEKEWQKDS 300
SMU69_05470      RTHDFQKKPIVNLLTWKHHLFVEKDYAKAKKSYDAAILFAQLTENINLRENLEKEWQKDS 300
SMU77_05920      RTHDFQKKPIVNLLTWKHHLFVEKDYAKAKKSYDAAILFAQLTENINLRENLEKEWQKDS 300
SMU81_05044      RTYDFQKKPIVNLLTWKYHLFVEKDYAKAKKSYDAAILFTQLTENINLRENLEKEWQKDS 300
SMU86_02980      RTHDFQKKPIVNLLTWKHHLFVEKDYAKAKKSYDAAILFAQLTENINLRENLEKEWQKDS 300
SMU93_06493      RTYDFQKKPIVNLLTWKHHLFVEKDYAKAKKSYDAAILFAQLTENINLRENLEKEWQKDS 300
SMU98_07471      RTHDFQKKPIVNLLTWKHHLFVEKDYAEAKKSYDAAILFAQLTENINLRENLEKEWQKDS 300
SMU104_00375     RTHDFQKKPIVNLLTWKHYLFVEKDYAKAKKSYDAAILFAQLTENINLRENLEKEWQKDS 300
SMU109_08657     RTHDFQKKPIVNLLTWKHHLFVEKDYAKAKKSYDAAILFAQLTENINLRENLEKEWQKDS 300
                 **:**************::*****:**:***********:********************

UA159            QNGT 304
SMU20_07973      QNGT 304
SMU21_00120      QNGT 304
SMU44_05115      QNGT 304
SMU52_07301      QNGT 304
SMU56_00582      QNGT 304
SMU57_07778      QNGT 304
SMU63_06475      QNGT 304
SMU69_05470      QNGT 304
SMU77_05920      QNGT 304
SMU81_05044      QNGT 304
SMU86_02980      QNGT 304
SMU93_06493      QNGT 304
SMU98_07471      QNGT 304
SMU104_00375     QNGT 304
SMU109_08657     QNGT 304
                 ****
```
