# Supplementary material for: Phenotypic Heterogeneity of Genomically-Diverse Isolates of Streptococcus mutans
Source: PLoS One. 2013 Apr 16;8(4):e61358. doi: 10.1371/journal.pone.0061358 (PMC3628994; doi:10.1371/journal.pone.0061358)
Supplement: Table S1 — Distribution of Type VII secretion genes among clinical isolates of S. mutans. (PDF) [file pone.0061358.s015.pdf]

Table S1. Distribution of Type VII secretion genes among clinical isolates of *S. mutans*

**Type VII SS**	**Smu44** ***e***	**Smu26** ***e***	**Smu80** ***c***	**Smu68** ***c***	**Smu97** ***c***	**Smu85** ***c***	**Smu108** ***c***	**Smu102** ***c***
EsxA/YukE	✔	✔	✔	✔	✔	✔	✔	✔
EsaA/YueB	✔	✔	✔	✔	✔	✔	✔	✔
EssA	✔	✔	✔	✔	✔	✔	✔	✔
EsaB/YukD	✔	✔	✔	✔	✔	✔	✔	✔
EssB/YukC	✔	✔	✔	✔	✔	✔	✔	✔
EssC/YukA	✔	✔	✔	✔	✔	✔	✔	✔
Duf3130*	✔	✔	✔	✔	✔	✔	✔	✔
Lmo0069	✔	✔	✔	✔				
EsaC-1	✔	✔		✔				
EsaC-2	✔	✔	✔		✔	✔	✔	
EsaC-3	✔	✔	✔	✔			✔	
EsaC-4	✔	✔	✔		✔			✔

*Conserved domain Duf3130 - Type VII secretion effector (SACOL2603 family), may be related to WGX100 proteins.
